# SARM1 is a multi-functional NAD(P)ase with prominent base exchange activity, all regulated bymultiple physiologically relevant NAD metabolites

**DOI:** 10.1016/j.isci.2022.103812

**Published:** 2022-01-25

**Authors:** Carlo Angeletti, Adolfo Amici, Jonathan Gilley, Andrea Loreto, Antonio G. Trapanotto, Christina Antoniou, Elisa Merlini, Michael P. Coleman, Giuseppe Orsomando

**Affiliations:** 1 Department of Clinical Sciences (DISCO), Section of Biochemistry, Polytechnic University of Marche, Via Ranieri 67, Ancona 60131, Italy; 2 John van Geest Centre for Brain Repair, Department of Clinical Neurosciences, University of Cambridge, Forvie Site, Robinson Way, CB2 0PY Cambridge, UK

**Keywords:** Biological sciences, Molecular physiology, Neuroscience

## Abstract

SARM1 is an NAD(P) glycohydrolase and TLR adapter with an essential, prodegenerative role in programmed axon death (Wallerian degeneration). Like other NAD(P)ases, it catalyzes multiple reactions that need to be fully investigated. Here, we compare these multiple activities for recombinant human SARM1, human CD38, and *Aplysia californica* ADP ribosyl cyclase. SARM1 has the highest transglycosidation (base exchange) activity at neutral pH and with some bases this dominates NAD(P) hydrolysis and cyclization. All SARM1 activities, including base exchange at neutral pH, are activated by an increased NMN:NAD ratio, at physiological levels of both metabolites. SARM1 base exchange occurs also in DRG neurons and is thus a very likely physiological source of calcium-mobilizing agent NaADP. Finally, we identify regulation by free pyridines, NADP, and nicotinic acid riboside (NaR) on SARM1, all of therapeutic interest. Understanding which specific SARM1 function(s) is responsible for axon degeneration is essential for its targeting in disease.

## Highlights

• Base exchange is a prominent, and sometimes completely dominant, SARM1 activity
• Physiologically relevant NMN:NAD ratios may regulate all of SARM1's multiple activities
• Physiological NADP may inhibit SARM1 more potently than NAD and via a distinct site
• NaR and VR both selectively inhibit SARM1 and are thus possible effectors or drug leads

## Graphical Abstract

**Figure:**
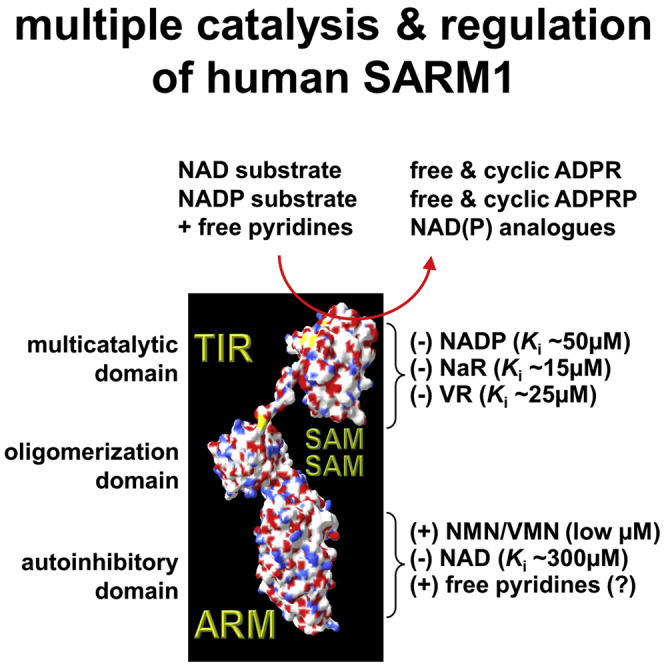

## Introduction

**Figure 1:**
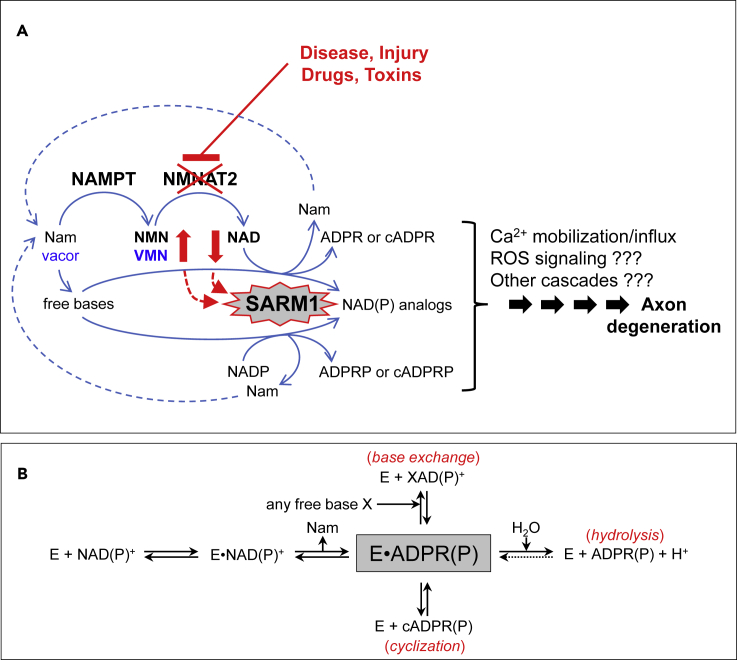
SARM1 is a central executioner of axon death via a multicomposite catabolic reaction typical of multifunctional NAD(P) glycohydrolases (A) Programmed axon degeneration is a widespread axon death mechanism driven by activation of SARM1 NAD(P)ase and prevented by its negative regulator NMNAT2, a short half-life enzyme essential to convert NMN into NAD. NMNAT2 forms together with the upstream enzyme NAMPT a key two-step pathway for NAD salvage in mammals that also provides metabolic conversion of the prodrug vacor into the neurotoxic, recently discovered, VMN intermediate. When NMNAT2 in axons becomes depleted or inactive, both NMN and VMN rise and NAD declines concomitantly; these fluctuations trigger SARM1 NAD(P)ase and, as shown here also base exchange, and thus initiate a signaling cascade that culminates into axon death. The early activation mechanism and key components of this process are highlighted in red and in blue for vacor toxicity. (B) Ordered Uni-Bi reaction mechanism of multifunctional NAD(P)ases (EC 3.2.2.6) like SARM1. From the substrate NAD(P), the pyridine moiety, Nam, is released first and then two distinct ADP ribosylated products arising from a single common intermediate (gray boxed). These may be ADPR(P) via classical hydrolysis or cADPR(P) via anhydrous cyclization. A third product XAD(P) represents any dinucleotides formed instead via transglycosidation (EC 2.4.99.20), a reaction of base exchange that replaces the pyridine moiety of the substrate with any related free base available.

SARM1 is an intracellular adaptor of Toll-like receptor (TLR) signaling and a prodegenerative enzyme with a central role in programmed axon death, or Wallerian degeneration (Coleman and Hoke, 2020; Osterloh et al., 2012). Wallerian degeneration occurs when axons are physically transected but it is now clear that the underlying programmed axon death mechanism is also activated by many toxins, gene mutations, or metabolic disruption (Conforti et al., 2014; Loring and Thompson, 2020). SARM1 is also constitutively hyperactivated by mutations associated with ALS (Bloom et al., 2021; Gilley et al., 2021). The crucial step common to many of these is loss of functional NMNAT2 from axons, an enzyme synthesizing the dinucleotide NAD from its precursor mononucleotide NMN. Specific NMNAT2 mutation or knockdown confirms that loss of this single protein is sufficient to cause axon degeneration or axon growth failure that is absolutely dependent on SARM1, with complete rescue when SARM1 is removed (Gilley et al., 2015, 2017; Huppke et al., 2019; Lukacs et al., 2019). Thus, loss of NMNAT2 lies upstream of the role of SARM1 in programmed axon death. Surprisingly, the rise in NMNAT2 substrate, NMN, when this enzyme is depleted, plays a particularly important role in activating the pathway (Di Stefano et al., 2015, 2017; Loreto et al., 2015, 2020). The unexpected discovery that SARM1 has intrinsic NADase activity (Essuman et al., 2017) then led to the finding that NMN activates SARM1 NADase (Zhao et al., 2019). NMN is now known to do this by binding an allosteric site on the SARM1 inhibitory ARM domain, and high levels of NAD compete to bind the same site, thereby opposing activation by NMN (Figley et al., 2021; Jiang et al., 2020; Sporny et al., 2020). VMN, a metabolite of the neurotoxin and disused rodenticide vacor, binds the same site and activates SARM1 even more potently (Loreto et al., 2021). Several negative regulators of SARM1 NADase have also been reported shown to act independently to the ARM domain, including the reaction product nicotinamide (Nam) (Bratkowski et al., 2020; Essuman et al., 2017) and some divalent cations (Loring et al., 2020a, 2020b). The current working model of the programmed axon death signaling pathway is shown in Figure 1A.

Another NAD(P) glycohydrolase, CD38, shows some catalytic similarity to SARM1 but no sequence similarity (Essuman et al., 2017; Loring and Thompson, 2020). CD38 NAD(P)ase does not appear to influence axonal degeneration (Sasaki et al., 2009) although it has roles in intracellular NAD homeostasis and calcium signaling (Chini, 2009) and is linked to other human diseases (Malavasi et al., 2008). SARM1 and CD38 thus control distinct fates in mammalian cells via intervention on related metabolism (Lee and Zhao, 2019), suggesting there may be subtle but important differences in the specific reactions they catalyze, categorised as NAD hydrolysis (EC 3.2.2.5), multifunctional enzymes catalyzing both synthesis and hydrolysis of cADPR (EC 3.2.2.6), or NADP hydrolysis and base exchange (EC 2.4.99.20) (Figure 1B). The structural basis of these distinct activities is at least partially understood (Liu et al., 2009); it involves an active site glutamate residue (Essuman et al., 2017; Graeff et al., 2001; Sauve et al., 1998), which transiently accommodates the ADPR moiety after hydrolysis of the β-N-glycosidic bond and release of Nam. The energy from this bond is conserved within the enzyme-bound complex allowing the direct formation of multiple products depending on which moiety is available or effectively joins the catalytic pocket. Use of water leads to hydrolysis, free pyridines lead to transglycosidation (base exchange), and in the absence of both there is cyclisation (Sauve et al., 1998). All of these activities have important roles in the synthesis of calcium mobilizers (Lee and Zhao, 2019; Sasaki et al., 2020): ADPR is the product of NAD hydrolysis, cADPR of its cyclisation, and base exchange is pivotal for the synthesis of NaADP from nicotinate and NADP (EC 2.4.99.20), of which there is no other known source in mammals (Berger et al., 2004).

In healthy axons, SARM1 exhibits a low, basal NAD(P)ase activity (Sasaki et al., 2020). Removing SARM1 greatly lowers basal levels of one NAD-derived product, cADPR, in several neural tissues but has only a modest, if any, effect on NAD levels (Gerdts et al., 2015; Gilley et al., 2015; Sasaki et al., 2020). Conversely, basal NAD is strongly elevated by removal of CD38 (Sasaki et al., 2009), but with limited effect on cADPR (Aksoy et al., 2006). This suggests that while CD38 is responsible for most NAD degradation under basal conditions, its primary product *in vivo* is not cADPR, while SARM1 is the main regulator of basal cADPR but other possibly greater activities of SARM1 remain possible. Base exchange by SARM1 has been reported very recently in live cells (Li et al., 2021) but, other than it being unaltered by NMN at pH 4.5 (Zhao et al., 2019), little is known about this activity.

The complex, tripartite domain structure of SARM1 contrasts with CD38 and its homolog ADP ribosyl cyclase from *Aplysia californica* (*Aplysia* cyclase) which lack separate regulatory domains (Liu et al., 2005; Prasad et al., 1996). SARM1 has an N-terminal ARM domain for auto-inhibition, two internal SAM domains for multimerization, and a C-terminal TIR domain for catalysis (Jiang et al., 2020; Sporny et al., 2020). TIR is an evolutionary ancient domain with many roles in innate immunity via protein interactions (Horsefield et al., 2019), which appears to have independently evolved as a NAD(P)ase (Essuman et al., 2018). The precise details of how activation of full-length SARM1 influences the TIR domain remain unclear, but TIR multimerization appears to be required for activation, either through allosteric alteration of the full-length oligomer (Bratkowski et al., 2020; Sporny et al., 2020) or by promoting dimerization artificially using a fused dimerizable domain (Gerdts et al., 2015) or macroviscogens like PEG (Loring et al., 2021). This also explains why at least some purified truncated forms of recombinant SARM1 were found to be inactive once purified unless they were assayed at high concentration or on-beads (Essuman et al., 2017; Horsefield et al., 2019; Loring et al., 2020a). SARM1's catalysis appears to be a product of functionally convergent evolution with homodimeric *Aplysia* cyclase and CD38 but within a multidomain protein architecture making it ideally suited as a proposed molecular switch (Bratkowski et al., 2020) from NAD(P) homeostasis in healthy axons to self-reinforcing NAD(P) decline in axons damaged beyond repair.

Despite the well-supported working model of programmed axon death (Figure 1A), many questions remain. One concerns the direct cause of axon death downstream of SARM1 activation. NAD depletion, leading to loss of ATP synthesis and of many other NAD-dependent activities (Yan et al., 2010), is an attractive candidate mechanism that has been widely assumed to be correct but direct evidence for this is lacking. Neurons and their axons can survive with remarkably low NAD levels (Figley et al., 2021), and while one SARM1 product, cADPR, has been partially excluded as a direct cause of axon death (Sasaki et al., 2020), there are others (see Figure 1A) that have not been investigated (Zhao et al., 2019). There are also other pyridine nucleotides degraded by SARM1 (Essuman et al., 2018). Moreover, while the NMN:NAD ratio is able to influence NAD turnover and cADPR synthesis by SARM1, its effect on these other activities especially at neutral pH remains unknown. It also remains unclear how SARM1 responds to modulation of either of these two key regulators when both are present at physiologically relevant levels, and whether other NAD-related metabolites also influence activity.

With all this in mind, we have compared the enzyme activities and regulation of hSARM1 to those of hCD38 and *Aplysia* cyclase. First, we identify a strong bias of SARM1 toward base exchanges both in enzyme assays using recombinant protein and in cultured DRG neurons, strongly suggesting this newly identified, multifunctional NAD(P)ase is also a key regulator of the potent Ca^2+^ mobilizer NaADP. Second, we show the ratio of NMN:NAD is a key determinant of SARM1 activity at physiologically relevant levels of each metabolite, an effect that requires the ARM domain and that at neutral pH is true for all known enzyme activities of SARM1. Third, we show there is also regulation by NADP through TIR domain interaction, again at physiologically relevant concentrations. Finally, we identify selective active site inhibition by nicotinic acid riboside (NaR) and analogs that could underlie new therapeutic strategies for axonopathies.

## Results

### Preliminary *in vitro* assays on selected multifunctional NAD(P)ases

**Figure 2:**
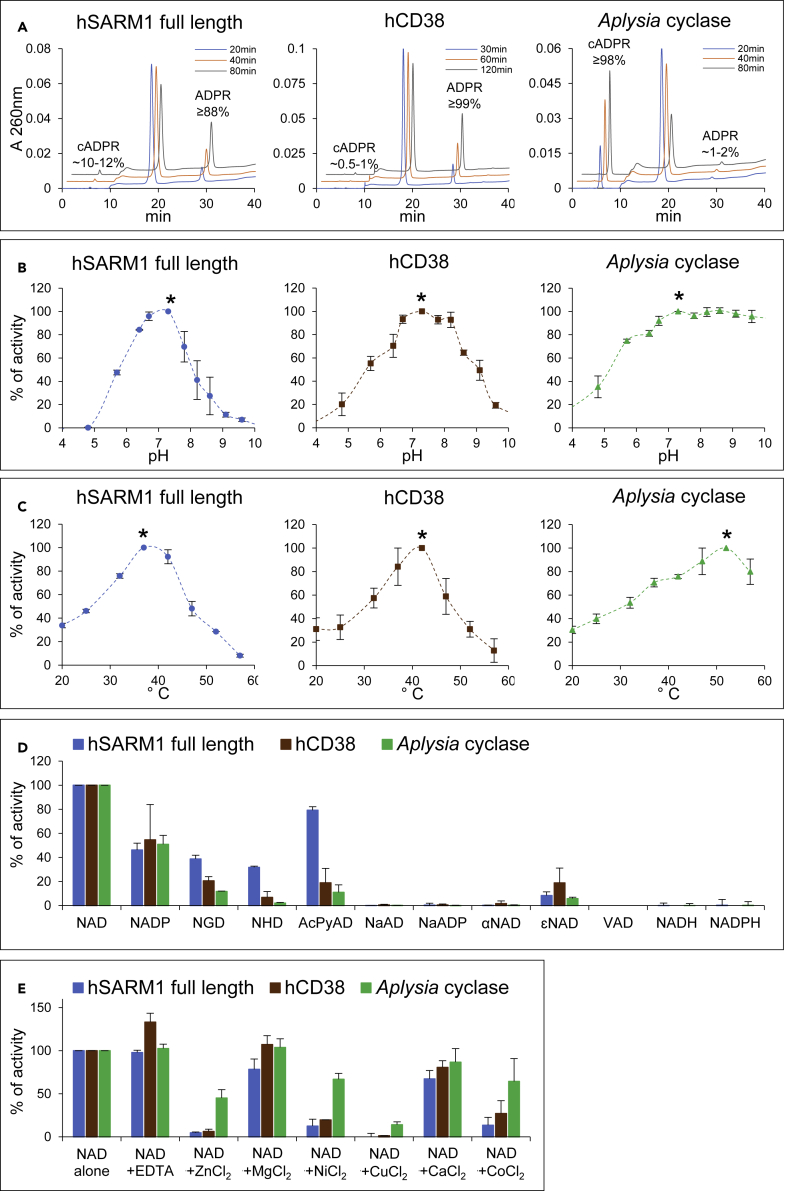
Preliminary characterization of selected multifunctional NAD(P)ases (A) C18-HPLC UV profiles from time-course analyses of the three indicated multifunctional NAD(P)ase enzymes studied, all assayed with 250 μM NAD at 25°C under similar rates of substrate consumption. Both ADPR and cADPR products accumulating into diverse proportions are highlighted while unmarked in between, the substrate NAD peak declines in parallel and proportionally by time. (B) pH studies carried out by HPLC assays in universal buffer (Tris/Bis-Tris/Na-acetate) for 1 h at 25°C using 0.25 mM NAD and 7 μg/mL hSARM1 or 0.5 mM NAD and 0.07 μg/mL CD38 or 6 mM NAD and 0.08 μg/mL *Aplysia* cyclase. Data are Mean ± SEM from n = 3 and are normalized to relative maxima of each curve (see asterisks). (C) Optimum temperatures. HPLC assays were set for 1 h using 0.25 mM NAD and 11 μg/mL hSARM1 or 0.5 mM NAD and 0.1 μg/mL CD38 or 2.5 mM NAD and 0.14 μg/mL *Aplysia* cyclase. Data are Mean ± SEM from n = 3 and are normalized as in (B). (D and E) Preferred substrates (D) and effects of metal ions (E). Various dinucleotides (250 μM each) or metal ions (1 mM each) were assayed at 25°C by HPLC for 2–6 h using 5.5 μg/mL hSARM1 or 0.1 μg/mL CD38 or 0.028 μg/mL *Aplysia* cyclase. Data are Mean ± SEM from n ≥ 2. Dinucleotide analogs indicated are: NGD, nicotinamide guanine dinucleotide; NHD, nicotinamide hypoxanthine dinucleotide; AcPyAD, 3-acetylpyridine adenine dinuclotide; NaAD, nicotinic acid adenine dinuclotide; NaADP, NaAD phosphate; αNAD, alpha-NAD; ϵNAD, nicotinamide 1,N^6^-etheno adenine dinucleotide; VAD, vacor adenine dinucleotide; NADH or NADPH, reduced NAD or NADP. See also Figures S1–S3.

We first established suitable conditions for commercial preparations of human CD38 (hCD38) and *A. californica* ADP ribosyl cyclase (*Aplysia* cyclase), and of human SARM1 full length (hSARM1) isolated from HEK cells (Figure S1), assaying the generation of multiple products by HPLC. The reaction rate for full-length hSARM1 showed a linear increase for concentrations up to at least ∼15 μg/mL (*i.e.* ∼0.2 μM, see STAR methods), thus confirming that our on-bead preparation was suitable for *in vitro* studies without further molecular crowding (Loring et al., 2021) or other interventions. We also verified activation under these conditions by NMN (Figure S2) and the total absence of catalytic activity for the previously reported enzyme-dead mutant E642A (Figure S3) (Essuman et al., 2017; Loreto et al., 2021; Zhao et al., 2019). Having established appropriate incubation times to determine initial rates with saturating NAD, we calculated specific activities for basal NADase through hydrolysis or cyclization combined of ∼0.02 U/mg for hSARM1, ∼7 U/mg for hCD38, and ∼50 U/mg for *Aplysia* cyclase, that were all consistent with expected values. Given the absence of free pyridines in these assays, we could individually measure the hydrolysis and cyclization activity of the ordered Uni-Bi mechanism (Berthelier et al., 1998) through the release of free or cyclic ADP ribosyl moiety (Figure 1B). There was clear divergence between the products of the three enzymes (Figure 2A), indicating a strong preference for cyclization by *Aplysia* cyclase and hydrolysis for hCD38 accounting for >98% of NAD consumption in both cases. hSARM1 also showed a preference for hydrolysis, with ∼90% NADase activity but a relatively high ∼10% of cyclization (Figure 2A). Varying NAD concentrations had no effect on these proportions (not shown).

We determined pH and temperature optima for each enzyme under comparable conditions. Maxima for hSARM1 and hCD38 were similar, within the physiological range of mammals (Figures 2B and 2C), whereas *Aplysia* cyclase was more tolerant of other conditions, probably reflecting its marine invertebrate origin, remaining active at basic pH or high temperatures (Figures 2B and 2C). Notably, changes to assay temperature or pH for each of the three enzymes influenced only the reaction rate, not the ratio of products formed as in Figure 2A (not shown). At pH 5 and below, where CD38 shows base exchange activity with a possible physiological role in lysosomes (Lee and Zhao, 2019; Nam et al., 2020), both hCD38 and hSARM1 showed NADase activities of ∼20% or less of their maxima (Figure 2B). All three enzymes at 25°C showed activity of ∼40% of their maxima (Figure 2C), so consistent with other recent reports (Essuman et al., 2017, 2018; Zhao et al., 2019), this temperature and pH 7.5 were chosen for subsequent experiments.

We then tested the use of alternative substrates and effects of divalent metal cations (Figures 2D and 2E) in view of the reported influence of zinc ions on SARM1 activity (Loring et al., 2020b) and also to determine assay conditions suitable to discriminate hSARM1 and hCD38 activity in mammalian tissue extracts, analogous to our reported isoform-specific assays for NMNAT activities (Orsomando et al., 2012). The physiological redox dinucleotides NAD and NADP were both among the preferred substrates of all three enzymes based on consumption rates. In contrast, there was negligible usage of either the corresponding reduced forms, NADH and NADPH, or the deamidated forms, NaAD and NaADP (Figure 2D). There was some consumption of NGD and NHD, which also occur naturally (Yaku et al., 2019), and of AcPyrAD and the artificial substrate ϵNAD. However, there was no activity toward αNAD or the vacor derivative VAD, despite prolonged incubations. Regarding the metal salts, magnesium or chloride counterions had no effect, while most other divalent cations tested, particularly copper and zinc, showed clear inhibition (Figure 2E). These results are consistent with previous reports (Essuman et al., 2018; Loring et al., 2020a, 2020b; Orsomando et al., 2000), but this side-by-side comparison reveals broader substrate use and greater divalent cation inhibition for hSARM1 compared to hCD38. The relative rates of hydrolysis and cyclization were also broadly similar to those with NAD (Figure 2A), except that hSARM1 showed a lower cyclization rate for NADP (≤1% of total NADP consumption) and CD38, a much higher cyclization rate for NGD (∼80% of total NGD consumption) (Graeff et al., 1994, 2001). These features, together with a selective inhibition by pyridine ribosides as described below, could be the basis of assays to distinguish CD38 and SARM1 in complex mixtures.

### Kinetics studies toward structural regulations on multidomain hSARM1

**Figure 3:**
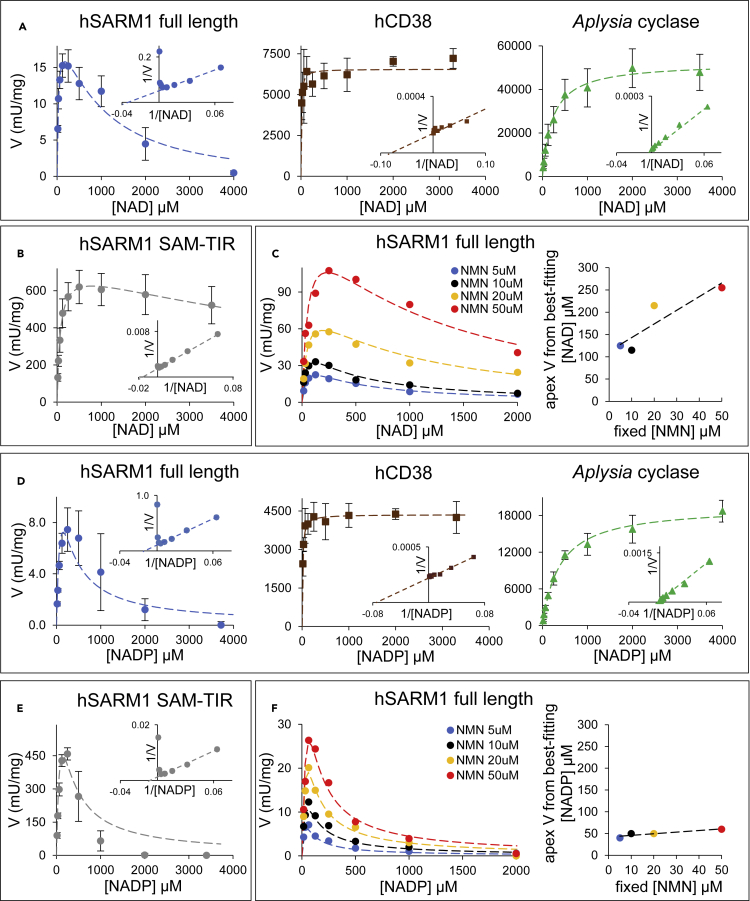
NAD and NADP kinetics by SARM1 in comparison to other multifunctional NAD(P)ases and under allosteric triggering by NMN (A) NAD kinetics of the three multifunctional NAD(P)ases. Initial rates were measured by HPLC using 3 μg/mL of hSARM1 or 0.05 μg/mL of CD38 or 0.02 μg/mL of *Aplysia* cyclase. Assays were carried out at 25°C for 30 min. Data are Mean ± SEM from n ≥ 3. Insets, double reciprocal plot analyses. Dotted lines, best fitting analyses carried out using the Equations 1 and 3 in STAR methods. The recalculated kinetic parameters are shown in Table 1. (B) NAD kinetics of the human SARM1 fragment SAM-TIR with constitutive NAD(P)ase that is not inducible by NMN because of the lack of the auto-inhibitory N-terminal regulatory domain ARM. HPLC assays were carried out at 25°C for 1 h using 2.75 μg/mL of enzyme per mix. Data are Mean ± SEM from n = 4. Inset, double reciprocal plot analysis. Dotted line, best fitting results from Equation 2 in STAR methods (see also recalculated parameters in Table 1). (C) NAD kinetics of hSARM1 full length (3 μg/mL per mix as in (A)) at increasing micromolar concentrations of the allosteric regulator NMN. Best fitting analysis was carried out on individual curves using Equation 3 in STAR methods to calculate the kinetic parameters shown in Table 1, and the relative curve maxima that were subsequently re-plotted on the flanking graph. Their linear relationship with the trigger amount in each curve indicates competition between NMN and NAD for opposing regulation of hSARM1 NADase. (D) NADP kinetics presented as for NAD in A above but done by assaying 14.6 μg/mL of hSARM1 or 0.05 μg/mL of CD38 or 0.07 μg/mL of *Aplysia* cyclase at 25°C for 60–120 min. Data are Mean ± SEM from n = 3. (E) NADP kinetics as in B above but done by assaying 1.9 μg/mL of SAM-TIR at 25°C for 1 h. Data are Mean ± SEM from n = 2. (F) NADP kinetics as in C above (3.7 μg/mL of hSARM1 full length per mix) at increasing micromolar concentrations of NMN. Flanking graph, best fitting maxima re-plotted showing NADP effects unrelated to allosteric triggering by NMN of hSARM1 NADPase. See also Figures S1 and S2.

We then studied kinetics of these three enzymes with respect to their physiological substrates NAD and NADP. The experimental initial rates from HPLC assays of our crude enzyme preparations, determined as described in the STAR methods, are shown in Figure 3 and kinetic constants arising from fitting of these data are listed in Table 1. Following immunoblot quantification of recombinant SARM1 proteins (Figure S1), the catalytic turnover (*K*_cat_) and efficiency (*K*_cat_/*K*_m_) were calculated (Table 2). In these conditions at least, hSARM1 catalyzes the hydrolysis of NADP a little less efficiently than its alternative physiological substrate, NAD.

**Table 1:** Kinetic constants obtained from best fitting of initial rates in Figure 3

	rep (n)	NAD substrate				rep (n)	NADP substrate			
		catalytic site			allosteric site		catalytic site			allosteric site
		*K*_m_ (μM)	*V*_max_ (mU/mg)	*K*_*is*_ (μM)	*K*_*i*a_ (μM)		*K*_m_ (μM)	*V*_max_ (mU/mg)	*K*_is_ (μM)	*K*_*ia*_ (μM)
hCD38	3	10.4 ± 2.90	6649 ± 124.7	–	–	3	11.7 ± 0.88	4376 ± 24.1	–	–
*Aplysia* cyclase	3	212 ± 13.7	51,353 ± 1237	–	–	3	384 ± 0.17	20,043 ± 724	–	–
hSARM1 full length	4	30.3 ± 5.18	22.4 ± 5.24	>8000	324 ± 24.6	3	66.9 ± 32.6	14.3 ± 3.14	132 ± 49.5	>8000
hSARM1 SAM-TIR	4	69.5 ± 2.31	723 ± 13.7	>8000	–	2	83.5 ± 11.9	582 ± 46.9	151 ± 34.1	–
+NMN 5μM	1	44.1	39.7	>8000	385.1	1	29.3	15.7	52.7	>8000
+NMN 10μM	1	34.2	54.1	>8000	414.9	1	45.9	29.9	57.4	>8000
+NMN 20μM	1	44.6	88.2	>8000	787.0	1	45.0	52.2	60.0	>8000
+NMN 50μM	1	56.1	161	>8000	1201.7	1	91.7	98.1	48.0	>8000

Best fitting was achieved by the equations described in STAR methods that were applied on replicates as indicated or on single curves at distinct NMN concentrations. “*K*_*is*_” and “*K*_*ia*_” are the affinity constants for the inhibition exerted, respectively, on the catalytic site on TIR domain and on the allosteric site on ARM domain of hSARM1. The calculated asymptotic rates are expressed per mg of protein in crude preparations.

**Table 2:** Steady-state kinetic parameters for wild-type human SARM1 and SAM-TIR

	NAD substrate			NADP substrate		
	*K*_m_ (μM)	*K*_cat_ (s^−1^)	*K*_cat_/*K*_m_ (M^−1^ s^−1^)	*K*_m_ (μM)	*K*_cat_ (s^−1^)	*K*_cat_/*K*_m_ (M^−1^ s^−1^)
hSARM1 full length	30.3 ± 5.18	0.030 ± 0.007	1000 ± 350	66.9 ± 32.6	0.019 ± 0.004	400 ± 250
hSARM1 SAM-TIR	69.5 ± 2.31	0.448 ± 0.009	6500 ± 350	83.5 ± 11.9	0.361 ± 0.029	4800 ± 700

Data values in Table 1 were re-elaborated for the indicated SARM1 species based on protein amounts quantified in their final preparations (see Figure S1).

In contrast to hCD38 and *Aplysia* cyclase, which both showed classical hyperbolic curves (Figure 3A) as reported (Berthelier et al., 1998; Hellmich and Strumwasser, 1991), hSARM1 did not. Its NADase activity rose to a maximum at 0.25 mM NAD and then progressively fell to almost zero at 4 mM (Figure 3A left). Measurements of NAD extracted from most mammalian tissues indicate levels between 0.2 and 1.0 mM (Mori et al., 2014) so the optimum is well positioned to respond to intracellular fluctuations of NAD such as those in circadian rhythms (Nakahata et al., 2009). This is particularly so in brain where basal levels are estimated at 0.3 mM (Mori et al., 2014). The absence of such strong inhibition on N-terminally truncated SARM1, consisting of only SAM and TIR domains (Figure 3B and Table 1), is consistent with recent reports of allosteric binding of NAD to the regulatory ARM domain (Figley et al., 2021; Jiang et al., 2020; Sporny et al., 2020), publications which reported widely varying inhibitory effects of NAD alone, or of blocking of NMN-induced activation by NAD. Importantly, our experiments used preparatory FPLC to guarantee the absence of contaminating impurities that often arise by degradation of NAD, including NMN and Nam, which will interfere with the measured response to variable NAD (see below).

As NMN and NAD exert reciprocal positive and negative regulation of SARM1 through competition at the same allosteric site (Figley et al., 2021), it is essential to determine SARM1 NADase kinetics close to the physiological levels of each of these regulators, and so far this has not been reported. Thus, we repeated our above analysis of NAD kinetics on full-length hSARM1 in the presence of increasing amounts of NMN (Figure 3C). In particular, we sought to determine the degree to which physiologically relevant levels of NAD could counter activation by physiological or pathological NMN, and how much falling NAD contributes to SARM1 activation around these physiological levels. We find that while inhibition by very high, non-physiological levels of NAD remains when hSARM1 is activated by NMN, NAD concentrations close to those in brain had only a marginal effect in countering activation by NMN as it rises from its physiological level of ca. 6 μM in brain (Mori et al., 2014) to more than double that in compromised axons (Di Stefano et al., 2015) (Figure 3C). In lesioned nerves, NAD levels approximately halve before axon fragmentation occurs (Di Stefano et al., 2015) although it is possible that a larger decline in axons is partially masked by relatively stable NAD in glia. Figure 3C shows that halving NAD from its normal nervous system level of ca. 300 μM marginally increases hSARM1 activity when NMN is close to basal levels (5 μM) but has decreasing effect as NMN rises. Indeed, if axonal NAD does drop by more than 50%, this could even lower hSARM1 NADase due to the lower availability of substrate. The shift in curve maxima to the right (Figure 3C) and increasing *K*_ia_ (Table 1) with increasing NMN are consistent with competition by NMN and NAD for the same regulatory site as reported (Bratkowski et al., 2020; Figley et al., 2021; Jiang et al., 2020; Sporny et al., 2020). These data strongly suggest that the rise in NMN when NMNAT2 is compromised is the principal determinant of hSARM1 activation, while fluctuations in NAD within the physiological or pathological range have limited effect.

SARM1 also has NADPase activity (Essuman et al., 2018). Considering the role of its reduced form in ROS buffering as well as many anabolic reactions, NADP loss is an additional candidate mechanism for SARM1-dependent axon death, but the response of SARM1 NADPase to NMN, or indeed to NADP itself, has not been reported. Thus, we performed similar analysis to those described above for NAD but now with variable NADP and NMN (Figures 3D-3F and Table 1). Again, we saw substrate inhibition for full-length hSARM1 NADPase (Figure 3D left), but in contrast to our findings with NAD this effect was fully retained in the absence of the ARM domain (Figures 3B and 3E). Physiologically relevant levels of NMN also strongly activated SARM1 NADPase (Figure 3F) with a potency very similar to that for SARM1 NADase (Figure S2), and the absence of a rightwards shift of the NADPase maxima, or of increasing *K*_is_ as NMN rises, further indicate that inhibition by NADP is not mediated by competition for the NMN binding site of the ARM domain. Instead, it is likely to interfere locally with TIR catalysis. Importantly, substrate inhibition by NADP shows a kinetic constant (*K*_is_) of just around 50 μM (Table 1), which again is close to best estimates of physiological NADP concentration in most mammalian cells (Trammell and Brenner, 2013) and tissues like brain (unpublished personal data).

In summary, these data show that (1) the basal catalytic efficiency of SARM1 is low for both NAD and NADP in comparison to other enzymes of the same class; (2) both NAD and NADP inhibit SARM1 TIR catalysis at high concentrations; (3) NADP is the more potent inhibitor (*K*_is_ ∼50 uM vs *K*_ia_ ∼300 uM), making this physiologically relevant even at its lower *in vivo* concentration; (4) inhibition by NAD is principally allosteric, in competition with NMN, whereas that by NADP is likely to occur at the catalytic TIR domain and is independent of NMN.

### Dominant and unique base exchange capability of hSARM1 NAD(P)ase

**Figure 4:**
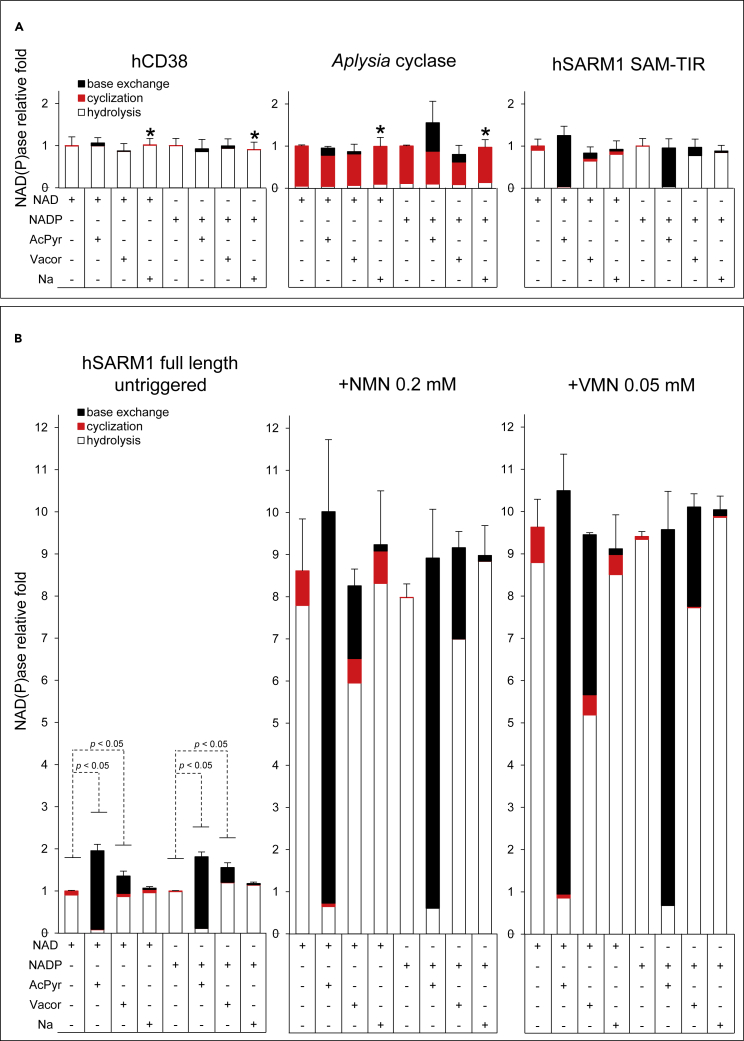
Base exchange reactions typically catalyzed by SARM1 and other multifunctional NAD(P)ases at neutral pH (A and B) Rates were measured in 50 mM HEPES/NaOH pH 7.5 at 25°C by HPLC using 0.05 μg/mL of CD38 or 0.1 μg/mL of *Aplysia* cyclase or 0.5 μg/mL of SARM1 SAM-TIR (upper panel A) or 7 μg/mL of SARM1 full length (bottom panel B). Both NAD and NADP substrates were fixed at 250 μM. The free bases 3-acetylpyridine (AcPyr) and nicotinic acid (Na) were added at 2 mM final. Vacor was added at 0.5 mM given its low solubility at physiological pH. The assay in B (left panel) is also shown in Supplementary (see Figure S4) and was replicated in the presence of two known allosteric regulators of SARM1, NMN 0.2 mM and VMN 0.05 mM (B, middle and right panels), leading both to a maximum triggering effect on SARM1 activity at this concentration as reported (Loreto et al., 2021). Multiple time stops from individual assays as above were analyzed for linearity, then extents of hydrolysis (white bars), cyclization (red bars), or base exchange (black bars) were calculated from each corresponding product as shown in Figure 1B and STAR methods. In detail, base exchanges led to form AcPyrAD from AcPyr, VAD from vacor, NaAD from Na in the presence of NAD or AcPyrADP from AcPyr, VADP from vacor, NaADP from Na in the presence of NADP (see Figure S4). Rates (Mean ± SEM, n = 2) are shown in histograms for comparison, referred to either NAD or NADP alone controls (arbitrarily fixed to 1). The whole dataset is also shown in Table S1. Asterisks (∗) indicate conditions where base exchange was below detection. See also Figures S3–S5, and Table S1

The base exchange activity of SARM1, using NADP and Na as substrates, was previously shown to exceed that of NAD hydrolysis or cyclization *in vitro* (Zhao et al., 2019). However, it was not further activated by NMN under the acidic assay conditions used to mirror the proposed NaADP synthesis activity of CD38 in lysosomes that also strictly depends on local NaAD as Na donor (Nam et al., 2020). SARM1, however, does not use NaAD to release Na, not at neutral pH at least (Figure 2D; Essuman et al., 2017; Gerdts et al., 2015), nor is thought to be a lysosomal enzyme (Lee and Zhao, 2019), so it is important to extend these studies to neutral pH and to other bases, and to directly compare base exchange and hydrolysis/cyclization rates using the same dinucleotide substrate. Thus, we characterised SARM1 base exchange activity for NAD or NADP paired with three different pyridine bases: 3-acetylpyridine (AcPyr), vacor (1-(4-nitrophenyl)-3-(pyridin-3-ylmethyl)urea), and nicotinic acid (Na) at pH 7.5 (Figure 4, Table S1).

Both hCD38 and *Aplysia* cyclase showed much lower base exchange reactions than hSARM1 under these conditions, and we confirmed previous reports about their undetectable activity at this pH with Na in particular (Figure 4A left, middle panels). In contrast, for both full-length SARM1 and its ARM-lacking fragment, base exchange was detectable for all three bases, including Na (Figures 4A right and 4B left), and we also found that the base exchange activity can displace hydrolysis and cyclization for both NAD and NADP to a degree dependent on the base used. The presence of AcPyr almost abolishes other reactions of SARM1 while vacor and Na leave both NADase and NADPase activities little altered although some extra VAD(P) or NaAD(P) are formed from exchanges, respectively, accounting for 20%–30% or 2%–3% of total products (Figures 4B left and S4, Table S1). For the full-length hSARM1 protein, overall use of both NAD and NADP also increases substantially in the presence of bases. All of these activities increased further still in the presence of either NMN 0.2 mM or VMN 0.05 mM, and leveled off at a plateau of ∼8- to 10-fold (Figure 4B middle, right panels). Note that the two distinct concentrations of triggers above were to guarantee a comparable, maximum triggering of SARM1 NADase, based on our previous report (Loreto et al., 2021). Thus, base exchange at neutral pH is regulated similarly to NAD(P) hydrolysis and cyclization but that activation by these regulators is not additive with the enhanced NAD or NADP loss caused by the free base alone.

The absolute dependence on the catalytic residue E642 (Figure S3), even in the presence of NMN, confirms that base exchange hSARM1 activity proceeds by the mechanism shown in Figure 1B. Lastly, we confirmed that all three bases above may also work together in mixture at micromolar concentrations (Figure S5), thus showing how multiple base exchanges may occur for SARM1 at the same time and without any apparent mutual competition among bases.

These data show that base exchange is largely unique to SARM1 in a physiological setting and can be dominant over the hydrolysis or cyclization of both NAD and NADP.

### SARM1-dependent base exchanges in DRG neurons

**Figure 5:**
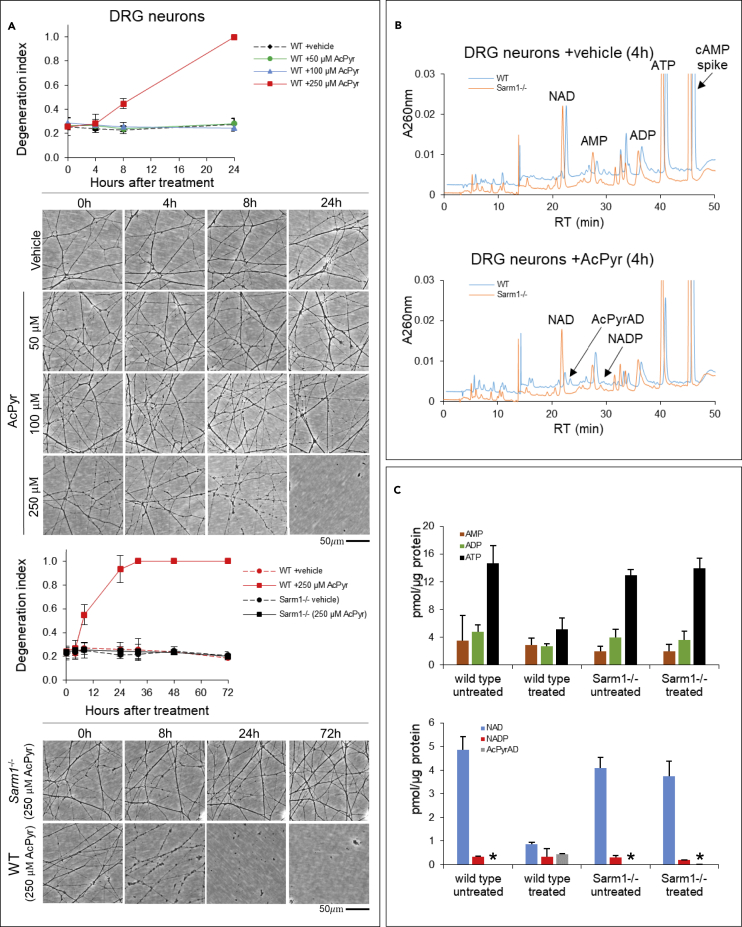
First evidence for SARM1-dependent base exchanges in DRG neurons (A) Quantification of the degeneration index and representative images of neurites from wild-type and *Sarm1*^−/−^ DRG explant cultures treated with 3-acetylpyridine (AcPyr) at the timepoints indicated (mean ± SD; n = 3). (B) Typical C18-HPLC UV profiles of the nucleotide extract obtained from wild-type and *Sarm1*^−/−^ DRG explant cultures following treatment for 4 h with AcPyr 250 μM or vehicle. The compounds of interest are indicated. (C) Measured levels of the different nucleotides extracted and analyzed as above (mean ± SD; n = 3). Data are normalized to the protein amount extracted in parallel. Asterisks (∗) indicate levels below detection.

We then explored whether SARM1-dependent generation of any of the above base exchange products could be seen directly in *ex vivo* cultured DRG neurons. Vacor and AcPyr, both of which are neurotoxins (Gallanosa et al., 1981; Desclin and Escubi, 1974), could be used. The toxic mechanism of each has recently been identified as activation of SARM1 by the corresponding mononucleotide, generated from these compounds within cells by enzymes of the physiological NAD synthesis pathway (Figure 1A) (Loreto et al., 2021; Sverkeli et al., 2021; Wu et al., 2021). The dose-response of DRG neurite degeneration to AcPyr was determined in order to select a condition shortly before degeneration occurs (Figure 5A). We chose a 4 h timepoint with 250 μM AcPyr, when neurites are still intact, to analyze metabolites by HPLC, following extraction from the wild-type or *Sarm1*^−/−^ DRG cultures. The non-physiological base exchange product AcPyrAD could be easily detected and measured in treated wild-type DRGs but not in treated *Sarm1*^−/−^ cultures, accounting for 0.446 ± 0.021 nmol of AcPyrAD formed per mg of protein (Figures 5B and 5C). Likewise, as we previously reported (Loreto et al., 2021), the dinucleotide VAD was also formed in DRG cultures from vacor. We can additionally confirm that this was found only in wild-type DRGs at a level of ∼0.14 nmol/mg following a sub-lethal dose of vacor 50 μM for 4 h (not shown). This indicates a strict, SARM1-dependent formation of base exchange products as expected within neuronal cells. Intriguingly, NMNAT2 may synthesize both VAD and AcPyrAD *in vitro* but not effectively into living neurons (Sverkeli et al., 2021; Wu et al., 2021), likely due to reduced catalytic efficiency (Buonvicino et al., 2018).

These data show that transglycosidation by SARM1 is a physiologically relevant activity, potentially even more than hydrolysis and cyclization. This opens to new important roles of SARM1 NAD(P)ase that have so far been little considered and indicate promising new therapeutic strategies (see Discussion).

### Selective inhibition by pyridine ribosides

**Figure 6:**
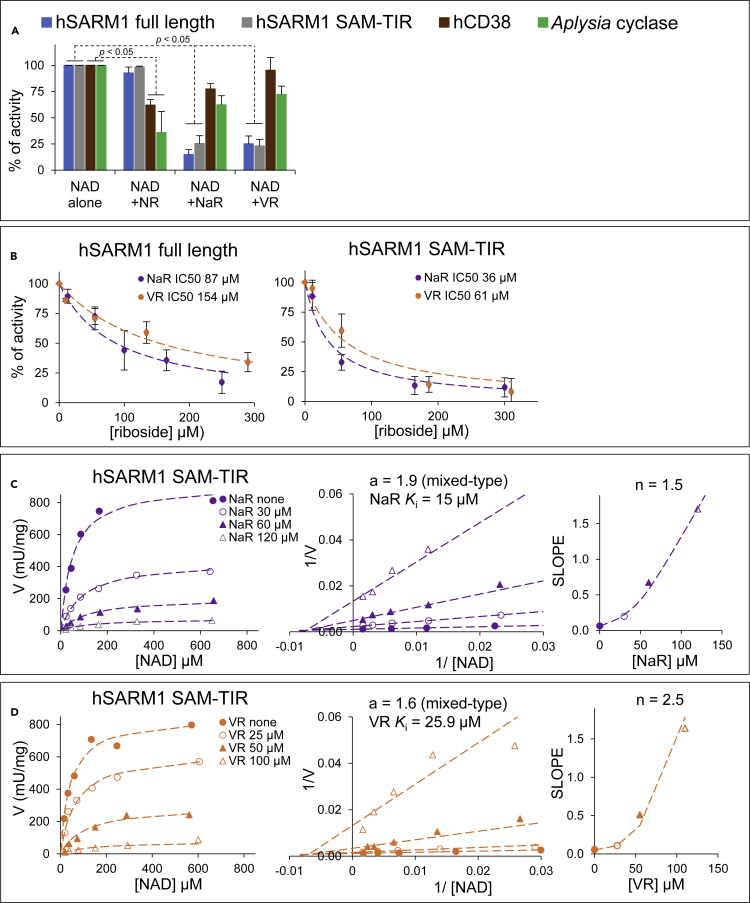
Selective inhibition by pyridine ribosides on multifunctional NAD(P)ase members (A) NADase inhibition at 200 μM fixed NR, NaR, or VR of the indicated enzyme species. Assays were carried out by HPLC at 25°C for 1 h using 0.25 mM of NAD and 13 μg/mL of SARM1 or 0.25 mM of NAD and 3.9 μg/mL of SAM-TIR or 0.25 mM of NAD and 0.1 μg/mL of CD38 or 1 mM of NAD and 0.02 μg/mL of *Aplysia* cyclase. Data are Mean ± SEM from n ≥ 4, normalized to NAD alone controls. (B) NADase inhibition at variable NaR or VR of SARM1 full length and SAM-TIR (assayed as in A above). Data are Mean ± SEM from n = 3. Dotted lines, best fitting analyses carried out using Equation 4 in STAR methods with the calculated IC50 values highlighted. (C and D) NADase inhibition kinetics under variable substrate NAD and at various fixed concentrations of either NaR or VR as indicated. SARM1 SAM-TIR (0.6 μg/mL in mix) was assayed by HPLC for 1–3 h at 25°C. The graph shows also Lineweaver-Burk plots (C and D middle) and slope replots (C and D right) indicating, respectively, inhibition type and *K*_i_ for both NaR and VR and the calculated number (n) of inhibitor molecules that bind to the enzyme. Best fitting analysis was done with Equation 5 in STAR methods.

The NAD(P)ase-enhancing effect on full-length hSARM1 exerted by free pyridine bases, and the opposing effects exerted by pyridine mono- and dinucleotides, prompted us to investigate also the effects of other pyridine moieties on this enzyme. So we assessed the pyridine ribosides, also in view of their membrane permeability that could support use as experimental tools and in drug development. We initially tested both natural and artificial pyridine ribosides at fixed concentration on the NADase activity of all three enzymes. To our surprise, we found that the two vitamin B3 precursors NaR and NR (Belenky et al., 2009; Bogan and Brenner, 2008; Bogan et al., 2009), exerted inhibitory effects but with opposing selectivity for hSARM1 *vs* both hCD38 and *Aplysia* cyclase (Figure 6A). Vacor derivative VR also strongly inhibited hSARM1 similar to NaR (Figure 6A). Preliminary IC50 measured at saturating NAD for NaR and VR on full-length hSARM1 were 87 and 154 μM, respectively, and inhibition of hSARM1 lacking its ARM domain was slightly stronger at IC50s of 36 and 61 μM (Figure 6B), indicating that this is not allosteric regulation through the ARM domain. Then, using the hSARM1 SAM-TIR fragment, we sought to determine the dissociation constant describing the binding affinity between the inhibitor and the enzyme and the kinetic mechanism of inhibition with results in the low micromolar range (*K*_i_ of 15 μM for NaR or of 25.9 μM for VR) and mixed-type inhibition for both NaR and VR (Figures 6C and 6D). We also obtained non-linear secondary plots of slopes and intercepts against concentrations of both NaR and VR, indicating concomitant binding at multiple sites for these inhibitors (Figures 6C and 6D right panels with the indicated “n” values >1).

These data indicate that (1) NaR and VR are good inhibitors of SARM1 catalysis with likely multiple interaction sites and no effects on the ARM-mediated allosteric communication of the protein; (2) NaR is a potential regulator of SARM1 *in vivo* considering its reported physiological level of 18 μM in wild-type yeast cells (Bogan et al., 2009), and, together with VR, is a promising lead structure for selective drug targeting of SARM1; and (3) NaR and NR could be used, together with the alternative substrates above, as a basis for discriminating activity of SARM1 and CD38 in mammalian tissue extracts.

## Discussion

Our data show that base exchange activity of SARM1 can be dominant over hydrolysis and cyclization of both NAD and NADP and always makes a contribution with each base we tested. It is particularly prominent with AcPyr and vacor both *in vitro* (Figure 4B) and in *ex vivo* cells (Figures 5B and 5C). We also show that all known SARM1 enzyme activities (NADase, NADPase, and base exchange for each dinucleotide) depend on the catalytically essential residue E642 and are regulated by physiologically relevant levels of NMN and NAD at neutral pH, in a manner that requires the N-terminal ARM domain. Below 250 μM NAD, which is close to physiological concentration, SARM1 NADase activity actually falls due to limited substrate availability, so when NMN rises and NAD falls after NMNAT2 loss (Di Stefano et al., 2015) we propose that increased NMN rather than lower NAD is the primary driver of increased SARM1 activity. Additionally, we describe three new inhibitors of SARM1, two of them present in normal physiology. NADP exerts substrate inhibition at physiologically relevant levels and both NaR and VR also inhibit. All three of these compounds are likely to act on the catalytic site in the TIR domain as their effects are independent of the allosteric site in the ARM domain.

The dominance of SARM1 base exchange over hydrolysis and cyclization depends ultimately on the specific base available but is generally consistent with the greater rate of base exchange previously reported when comparing hydrolysis or cyclization of an NAD substrate with base exchange involving NADP at two different pH values (Zhao et al., 2019). It could also help to explain why during the first 4 h following axotomy in DRG primary cultures the molar rise in axonal cADPR is 7- to 8-fold less than the molar fall in NAD, with very little of another alternative product ADPR being detected (Sasaki et al., 2020). Thus, while different cADPR measurements in *Sarm1*^−/−^ and wild-type axons, and the SARM1-dependent increase in cADPR after axotomy (Sasaki et al., 2020), clearly show that NAD cyclization is one relevant physiological and pathological activity of SARM1, it is unlikely to be the only one. Base exchange by SARM1 could be the primary driver of NAD decline, particularly in the presence of a base such as 3-acetylpyridine that strongly favors this activity, and also likely represents an important physiological activity of SARM1 under normal conditions.

In addition to confirming a previous report that SARM1 base exchange activity can catalyze the formation of the calcium-mobilizing agent NaADP, we show that this activity is induced by NMN and occurs at neutral pH not just at pH 4.5. This is important because SARM1 is not known to be a lysosomal enzyme and certainly also localizes elsewhere in non-acidic compartments (Gerdts et al., 2013; Osterloh et al., 2012). Thus, we propose SARM1 is a source of cytosolic NaADP in mammals, alongside the recently reported lysosomal source involving CD38 (Nam et al., 2020), but using Na rather than NaAD at neutral pH. This is consistent with the observation that *Cd38*^−/−^ mice do not have lower levels of this metabolite (Schmid et al., 2011; Soares et al., 2007) even if CD38 can generate NaADP in specific *in vitro* conditions (Guse and Lee, 2008). Importantly, this also means that the reported absence of any detectable influence of cADPR on axon survival (Sasaki et al., 2020) does not exclude a key role for calcium mobilization as the SARM1-dependent activity driving axon degeneration, consistent with the observed SARM1-dependent rise in calcium (Loreto et al., 2015). The roles of NaADP and other potential products of base exchange have been relatively neglected by the focus on NAD loss but our findings here show the vital importance of taking a wider perspective of SARM1 activities.

Another SARM1 activity with strong potential to cause axon degeneration is NADP hydrolysis (or cyclization) (Essuman et al., 2018). The reduced form of this coenzyme, NADPH, has a crucial role in ROS buffering, so SARM1-dependent NADPase puts axons at considerable risk of ROS-induced degeneration, and indeed activation of Wallerian degeneration by vincristine does cause an increase in ROS (Press and Milbrandt, 2008). Importantly, therefore, we now show that SARM1 NADPase, as well as NADase and base exchange at neutral pH, is regulated by the NMN:NAD ratio at physiologically relevant levels of both metabolites. Thus, each, or any combination of these activities could underlie the NMN-dependent degeneration we reported previously (Di Stefano et al., 2015, 2017).

Our data also have many implications for the development of therapies for axonopathies. Animal and cell culture data strongly implicate SARM1-dependent axon loss in some peripheral neuropathies, glaucoma, Parkinson disease, and other conditions (Coleman and Hoke, 2020; Krauss et al., 2020; Loring and Thompson, 2020), and human genetic studies support its involvement in rare axonopathies involving *NMNAT2* mutation (Huppke et al., 2019; Lukacs et al., 2019) and in ALS (Bloom et al., 2021; Gilley et al., 2021). This, together with the complete rescue of axons by SARM1 removal when the pathway is very specifically activated (Gilley et al., 2017; Loreto et al., 2021), has led to considerable focus on inhibiting and knocking down SARM1 as a therapeutic strategy (Gould et al., 2021; Li et al., 2021; Loring and Thompson, 2020; Sasaki et al., 2020). Many studies are focusing on blocking the NADase activity specifically but our data now suggest the importance of targeting NADPase and base exchange too. This is important because the most effective drugs will come from targeting the right enzyme activity, indeed small molecules are already known to influence different SARM1 activities in different ways (Li et al., 2021; Zhao et al., 2019).

New physiological inhibitors of SARM1 activity identified in this study indicate potential novel routes to block SARM1. In particular, substrate inhibition by physiologically relevant levels of NADP suggests that strategies to elevate NADP *in vivo* could be useful. The riboside NaR also has considerable potential on account of its membrane permeability and tolerance by normal physiology, and indeed inhibition of SARM1 may have contributed to its protective role in a reported combinatorial treatment with the NAMPT inhibitor FK866 (Liu et al., 2018a). However, while NaR is a normal physiological metabolite (Bogan et al., 2009; Carpi et al., 2018; Kulikova et al., 2015), its levels in most mammalian tissues, including neurons, are uncertain. The deamidated branch of NAD biosynthesis in mammals is a minor component (Mori et al., 2014) and normal NaR levels are thought to be marginal compared to the historical B3 vitamins Nam and Na and the more recently studied NR (Bieganowski and Brenner, 2004; Bogan and Brenner, 2008). Nonetheless, our *in vitro* finding of a *K*_i_ value in the low micromolar range, lower indeed than *K*_m_ for both substrates (see Figures 6C and 6D, Table 1), supports the potential for regulation(s) by NaR *in vivo* under specific conditions, or following exogenous application. Understanding the structural basis of the differential inhibition by NaR, VR, and the inactive NR, will now be a good basis for rational drug design.

Finally, there are several ways in which the preference by SARM1 for base exchange may be exploited as a basis for therapy. This finding suggests that base exchange involving some free bases could contribute substantially to NAD loss, so identifying and lowering the level of such bases could be effective. Our data also indicate a need to reconsider the proposed inhibitory effect of nicotinamide (Nam) on SARM1 NADase (Bratkowski et al., 2020; Essuman et al., 2017). In simple assays of the effect of Nam on NAD hydrolysis, it would be impossible to distinguish inhibition from competition between Nam and water in the final step, the former simply switching the incumbent Nam on NAD for a new, identical group and thus appearing to have done nothing. These could only be distinguished using isotopic labeling of substrates. Such an action could also be the basis of the observed therapeutic or preventative benefit of Nam supply in several conditions involving axon loss (Anderson et al., 2013; Hui et al., 2020), so if this could be confirmed as the mechanism there may be ways to boost it further.

In summary, we show that SARM1 is a multidomain NAD(P)ase regulated by pyridine mono- and dinucleotides but also by corresponding ribosides and free bases, and that, as shown in Figure 1A, its base exchange may have considerably greater relevance in SARM1-dependent axon death than previously considered. In some circumstances, it is a dominant activity of SARM1 *in vivo*; it is regulated like other SARM1 enzyme activities by physiological levels of NMN and NAD, consistent with a role in NMN-dependent axon death (Di Stefano et al., 2015, 2017); it is likely to be a major physiological source of calcium mobilizing signal NaADP; and its manipulation by dietary or pharmacological means has important potential for prevention and therapy of axonal disorders. We also report inhibition of SARM1 by NADP and NaR, with the membrane permeability and physiological tolerance of the latter in particular a property that lends itself to effective therapy.

### Limitations of the study

The precise levels of the physiological pyridine metabolites used in this study are unknown, and are likely to differ between cell types, between physiological and pathological states, and between subcellular compartments (Liu et al., 2018b). Thus, translation of our *in vitro* data into *in vivo* predictions depends on these unknown levels but the ranges we studied are close to those relevant to some physiological and pathological states. For instance, our previous study (Mori et al., 2014) was our reference for basal axonal NAD and NMN levels, in close agreement with others (Cuny et al., 2021; Di Stefano et al., 2017; Kim et al., 2020; Parker et al., 2020) and we also showed that NMN rises and NAD falls in damaged nerves (Di Stefano et al., 2015). Also presently unknown are the contribution (if any) from glia to pyridine metabolism in neuronal cells, and any modulating effects of SARM1 interacting proteins.

## STAR★Methods

### Key resources table

**Table:** 

REAGENT or RESOURCE	SOURCE	IDENTIFIER
**Antibodies**		

Mouse monoclonal anti-FLAG M2	Merck	Cat#F3165; RRID AB_259529

**Chemicals, peptides, and recombinant proteins**		

AcPyr (3-acetylpyridine)	Merck	Cat#A21207
Na (nicotinic acid)	Merck	Cat#N4126
NaR (nicotinic acid riboside)	This paper (made enzymatically from NaAD)	N/A
NR (nicotinamide riboside)	Tru Niagen®	250 mg capsules
NaAD (nicotinic acid adenine dinucleotide)	Merck	Cat#N4256
NAD (nicotinamide adenine dinucleotide)	Merck	Cat#N1511
NADP (nicotinamide adenine dinucleotide phosphate)	Merck	Cat#N5755
NaADP (nicotinic acid adenine dinucleotide phosphate)	Merck	Cat#N5655
Vacor (Pyrinuron)	Greyhound Chromatography	Cat#N13738
VMN (vacor mononucleotide)	Buonvicino et al., 2018; Loreto et al., 2021	N/A
VAD (vacor adenine dinucleotide)	Buonvicino et al., 2018; Loreto et al., 2021	N/A
VR (vacor riboside)	This paper (made enzymatically from VMN)	N/A
*Aplysia californica* ADP ribosyl cyclase	Merck	Cat# A8950
Calf Intestine Alkaline Phosphatase (CIAP)	Applichem	Cat#A3810
*Crotalus adamanteus* Phosphodiesterase I (PDE-I)	Merck	Cat#P3134
Human CD38 (fragment Val43-Ile300 + His tag)	R&D Systems	Cat#P28907
Human SARM1 (full length + FLAG tag)	Gilley et al., 2021; Loreto et al., 2021	N/A
Human SARM1 E642A (full length + FLAG tag)	Gilley et al., 2021; Loreto et al., 2021	N/A
Human SAM-TIR (fragment Val409-Thr724 + FLAG tag)	Gilley et al., 2021; Loreto et al., 2021	N/A

**Critical commercial assays**		

NAD/NADH-Glo^TM^ Assay	Promega	Cat#G9071

**Experimental models: Cell lines**		

Human: HEK 293T	ATCC clone 17	Cat#CRL-11268; RRID CVCL_1926
Mouse: DRG neurons wild-type (from C57BL/6J)	This paper (made from E13.5-E14.5 embryos)	RRID IMSR_JAX:000664
Mouse: DRG neurons *Sarm1*^-/-^	This paper (made from E13.5-E14.5 embryos)	RRID MGI_3765957

**Software and algorithms**		

Fiji	Free open source	http//fiji.sc; RRID:SCR_002285
R v4.1.1-win 2021	Free open source	https://www.R-project.org/

### Resource availability

#### Lead contact

Further information and requests for resources and reagents should be directed to and will be fulfilled by the lead contact, Giuseppe Orsomando (g.orsomando@staff.univpm.it).

#### Materials availability

This study did not generate new unique reagents

#### Data and code availability

All data reported in this paper will be shared by the lead contact upon request. This paper does not report original code. Any additional information required to reanalyze the data reported in this paper is available from the lead contact upon request.

### Experimental model and subject details

Dorsal Root Ganglia (DRG) were dissected from C57BL/6J (RRID IMSR_JAX:000664) or *Sarm1*^-/-^ (RRID MGI_3765957) E13.5-E14.5 mouse embryos. Explants were cultured in 35 mm tissue culture dishes pre-coated with poly-L-lysine (20 μg/ml for 1 h; Merck) and laminin (20 μg/ml for 1 h; Merck) in Dulbecco's Modified Eagle's Medium (DMEM, Gibco) with 1 % penicillin/streptomycin (Thermo Fisher Scientific), 33 ng/ml 2.5S NGF (Thermo Fisher Scientific) and 2% B27 (Gibco). 4 μM aphidicolin (Merck) was used to reduce proliferation and viability of small numbers of non-neuronal cells (Loreto and Gilley, 2020). Human embryonic kidney (HEK) 293T cells (ATCC clone 17, CRL-11268, RRID CVCL_1926) were cultured in DMEM with 4.5 g/L glucose and 110 mg/L sodium pyruvate (PAA), supplemented with 2 mM glutamine (Invitrogen), 1% penicillin/streptomycin, and 10% fetal bovine serum (PAA).

### Method details

#### Reagents

Vacor (Pyrinuron N-13738) was purchased from Greyhound Chromatography (UK) and used to generate VMN and VAD enzymatically *in vitro* as reported (Buonvicino et al., 2018; Loreto et al., 2021). The ribosides VR and NaR were also obtained enzymatically *in vitro* from digestion of VMN and NaAD, respectively (see next paragraph). The riboside NR was prepared from Tru Niagen® capsules by dissolving the contents in water and filtering. Other chemicals were all at the highest purity level available and used without further treatment except for NAD, NADP and NaADP that were purified by IEC-FPLC prior to use (see “Purification of contaminated pyridine dinucleotides”).

#### Preparation of vacor riboside (VR) and nicotinic acid riboside (NaR)

VR was obtained by de-phosphorylation of VMN using Calf Intestine Alkaline Phosphatase (CIAP, Applichem A3810, resuspended at ∼50 mg/ml in 100 mM Tris/HCl, pH 9, 100 mM NaCl). NaR was obtained by simultaneous de-phosphorylation/pyrophosphorolysis of NaAD using the CIAP above plus the *Crotalus adamanteus* Phosphodiesterase I (PDE-I, Merck P3134, resuspended at ∼10 mg/ml in 100 mM Tris/HCl, pH 9, 100 mM NaCl). Briefly, a reaction mixture (A) for VR and a reaction mixture (B) for NaR were set in the resuspension buffer above containing 15 mM MgCl_2_ and either 0.4 mM VMN and 2 mg/ml CIAP (∼20 U/mg at 25°C) or 0.425 mM NaAD and 2 mg/ml CIAP plus 0.2 mg/ml PDE-I (∼0.02 U/mg at 37°C). Mixtures (A) and (B) of 0.5 ml and 1 ml final volumes respectively were incubated for 1 h and 2 h at 37°C. At the end, they were boiled, centrifuged and loaded on C18-HPLC (Varian, 90 Å, 5 μm). Preparative runs were performed at two distinct temperatures under elution gradients obtained in volatile buffers (see Figure S6 upper panel). So, VR eluted at ∼50% acetonitrile after heating the column at 50°C, whereas NaR eluted at less than 5% acetonitrile after cooling the column at 12°C. Both ribosides were collected, quantified spectrophotometrically by the ϵ_340nm_ of 17.8 mM^−1^ cm^−1^ (VR) or the ϵ_260nm_ of 4.0 mM^−1^ cm^−1^ (NaR), frozen and lyophilized. Dry pellets were stable at −80°C for months. Typical yields were ∼150 nmol of VR, *i.e.* ∼75% of the original ∼0.2 μmol VMN in mixture (A), or ∼300 nmol of NaR, *i.e.* ∼70% of the original ∼0.43 μmol NaAD in mixture (B). Lyophilized VR and NaR resulted pure after resuspension and re-injection on HPLC.

#### Purification of contaminated pyridine dinucleotides

Stocks in water of NAD (Merck N1511), NADP (Merck N5755), and NaADP (Merck N5655) degraded often after repeated freezing/thawing, thus generating interfering contaminants such as NMN or Nam. We therefore cleaned them up as follows before use. Briefly, an ion-exchange chromatography (IEC) was performed on AKTA Purifier (FPLC) onto a TSK DEAE column (Tosoh, 4.6 × 250 mm). The IEC-FPLC equilibration was at 1 ml/min and 25°C, followed by salt gradient elution (see Figure S6 bottom). Typically, 1-2 micromoles per run of NAD or NADP or NaADP were injected, eluted and pooled from multiple runs, then diluted and quantified by UV absorption (ϵ_260nm_ of 18 mM^−1^ cm^−1^), frozen and lyophilized. After resuspension and HPLC re-injection, all three dinucleotides resulted pure at ≥99.8% and were used immediately.

#### Proteins and long-term storage

Human CD38 (P28907, a soluble fragment Val43-Ile300 with 6x-His tag at C terminus) was purchased from R&D Systems (USA). It was diluted to final 0.01 mg/ml in 100 mM HEPES/NaOH buffer, pH 7.5, BSA 0.1 mg/ml and stored at −80°C in aliquots. *Aplysia californica* ADP ribosyl cyclase was purchased from Merck (A8950), resuspended at 1 mg/ml in Tris/HCl 50 mM, pH 7.0 and stored at −20°C in aliquots. Human recombinant SARM1 full length proteins (wild-type and E642A mutant) and wild type SAM-TIR fragment Val409-Thr724 were expressed in HEK 293T cells with a Flag-tag at C terminus. The transfection, expression and purification protocols were as previously described (Gilley et al., 2021; Loreto et al., 2021). At the end, the on beads preparations were resuspended in PBS containing 1 mg/ml BSA (included to protect proteins during freezing), using ∼150 μL per ml of the immunoprecipitation volume for SARM1 full length species or ∼50 μl for SAM-TIR (due to lower yield resulting from higher enzymatic activity). Resuspended immunoprecipitated proteins were finally stored at −80°C in aliquots.

#### Immunoblotting and quantification of SARM1 recombinant species

Bead suspensions of immunoprecipitated proteins were diluted 1 : 16 in 2x SDS-PAGE loading buffer and heated to 100°C for 3 min. Samples (10 μL per well) were resolved on 4-20% gradient gels (Bio-Rad) alongside a dilution series of pure recombinant SARM1 of known concentration (kindly provided by AstraZeneca), before being transferred to Immobilon-P membrane (Millipore). Blots were blocked in TBS (20 mM Tris pH 8.3, 150 mM NaCl) containing 5% skim milk powder for 30 min at RT before being incubated overnight at 4°C with a rabbit polyclonal antibody raised against the SAM domains of human SARM1 (kindly provided by AstraZeneca) in TBS containing 0.05% Tween 20 (TBST) and 5% milk. After 3 × 10 min washes in TBST, blots were incubated for 1-2 h at RT with an anti-rabbit HRP-conjugated secondary antibody (diluted 1 : 3,000, Bio-Rad) in TBST with 5% milk. After 3 × 10 min washes in TBST and one rinse in TBS, blots were incubated with Pierce ECL Western Blotting Substrate (Thermo Fisher Scientific) and imaged using an Alliance chemiluminescence imaging system (UVITEC Cambridge). Relative band intensities on captured digital images were determined from areas under histogram peaks using Fiji software (http:fiji.sc) and concentrations of SARM1 and SAM-TIR in the bead suspension were calculated from a standard curve generated from the dilution series of a recombinant SARM1 as shown (see Figure S1). To confirm the reliability of this quantification method and the overall purity of the preparations ∼20 times the amount of each sample were resolved by SDS-PAGE as above and gels were stained with GelCode Blue reagent (Thermo Fisher Scientific). Relative band intensities of the purified proteins were found to be comparable.

#### NADase assays

hSARM1 species were routinely assayed by the NAD/NADH-Glo^TM^ Assay (Promega) while both *Aplysia* cyclase and CD38 were assayed by fluorometry using ϵNAD (Orsomando et al., 2000). All NAD(P)ases were also assayed by HPLC in mixtures containing 0.02-15 μg/mL protein and 0.015-4 mM NAD or NADP as substrates, dissolved in 50 mM HEPES/NaOH, pH 7.5. Other reagents used in addition to, or in place of those above are detailed in text or in figure legends, for example metal ions, free bases, dinucleotide analogues, mononucleotides, and ribosides. Assays were all carried out at 25°C except for temperature studies. Reactions were stopped by HClO_4_/K_2_CO_3_ and analysed by C18-HPLC as reported (Mori et al., 2014). Only the acid labile NADH and NADPH were treated with 1 N NaOH and neutralized using 1:1 vol of 1.73 M KH_2_CO_3_ while the highly hydrophobic metabolites vacor, VAD, VMN and VR, were analysed by a modified C18-HPLC method (Buonvicino et al., 2018). Reaction products were quantified from peak areas (see Figure 2A) based on coelution with standards (except for cyclic products from NAD analogues that were not available and were attributed based on UV-spectra and retention times as predicted). Rates were calculated as follows. Hydrolysis and cyclization were measured from product generation from the respective individual dinucleotide substrate used, e.g., free and cyclic ADP-ribosyl moieties from NAD (Figure 1B). A basal value for hydrolysis/cyclization was taken as sum of these latter two products. In addition to that value, base exchange or transglycosidation was calculated from the expected dinucleotide analog formed in the presence of the corresponding free base, e.g., AcPyrAD from AcPyr and NAD. The measured level of overall formed products matched in all cases the dinucleotide substrate consumed and the corresponding pyridine base released, according to the reaction scheme (Figure 1B). Furthermore, enzyme amounts forming 1 μmol/min of overall products above were referred to to as single units (U) of activity and initial rates were always considered, for example until a maximum of 20% of the original substrate had been consumed and all products were still accumulating at a nearly linear rate.

#### Temperature and pH studies

Temperature-dependence was carried out in buffer as above while pH studies were carried out in universal buffer 50 mM, pH 3-11 (167 mM Tris, 167 mM Bis-Tris, 167 mM sodium acetate). Stocks of 10X universal buffer were adjusted to each pH with 5 N HCl and diluted in the assay. Controls without enzyme were run in parallel to correct for any non-enzymatic NAD degradation at pH ≥ 8.0 or at the respective temperatures studied.

#### Kinetics and inhibition studies

Kinetics were performed by varying NAD or NADP from 15 μM to 4 mM. Their stocks were free from NMN, Nam or any other HPLC-detectable contaminant (see “Purification of contaminated pyridine dinucleotides” and Figure S6 bottom ), and controls were run in parallel to correct for any potential background. The kinetic parameters were calculated from initial rates obtained from replicates via best-fitting analysis using the following Equation 1 for CD38 and *Aplysia* cyclase, or Equation 2 for hSARM1 SAM-TIR, or Equation 3 for hSARM1 full length

(Equation 1) $$\text{V }=\frac{{\text{V}}_{\text{max}}\left[\text{S}\right]}{{\text{K}}_{\text{m}}+\left[\text{S}\right]}$$

(Equation 2) $$\text{V }=\frac{{\text{V}}_{\text{max}}\left[\text{S}\right]}{{\text{K}}_{\text{m}}+\left[\text{S}\right]\left(1+\frac{\left[S\right]}{Kis}\right)}$$

(Equation 3) $$\text{V }=\frac{{\text{V}}_{\text{max}}\left[\text{S}\right]}{\left({\text{K}}_{\text{m}}+\left[\text{S}\right]\left(1+\frac{\left[S\right]}{Kis}\right)\right)\left(1+\frac{\left[S\right]}{Kia}\right)}\;$$

where, for both NAD and NADP, *K*_is_ represents the affinity constant for inhibition on the catalytic TIR site and *K*_ia_ the affinity constant for inhibition on the allosteric ARM site. Kinetics of full length SARM1 with both NAD and NADP was also performed at increasing fixed concentrations of NMN ranging from 5 μM to 50 μM. In that case, the resulting single curves were fit to Equation 3 as above to evaluate any competition between NMN and the substrate used (see Table 1).

Inhibition by ribosides was assayed by HPLC initially at fixed 200 μM of each NR, NaR or VR and optimized NAD concentrations for the different enzymes (see Figure 6 legend). Then, at same NAD concentration as above, IC50 was assessed by varying NaR or VR from 10 μM to 300 μM, and calculated via best fitting of initial rates vs no inhibitor controls (SARM1 activity %) to the Equation 4 below.

(Equation 4) $$\text{SARM}1\;\text{activity\%}=\frac{1}{1+\frac{\left[\text{I}\right]}{\text{IC}50}}$$

Finally, kinetic constants and mechanism of inhibition were assayed under variable NAD 15-600 μM (where substrate inhibition is negligible) and fixed NaR or VR at various concentrations ranging from 25 μM to 120 μM, and the initial rates obtained were fit to the rate Equation 5 below (valid as long as substrate inhibition is negligible)

(Equation 5) $$\text{V }=\frac{{\text{V}}_{\text{max}}\left[\text{S}\right]}{{\text{K}}_{\text{m}}\left(1+{\left(\frac{\left[\text{I}\right]}{{\text{K}}_{\text{i}}}\right)}^{n}\right)+\left[\text{S}\right]\left(1+{\left(\frac{\left[\text{I}\right]}{{\text{aK}}_{\text{i}}}\right)}^{n}\right)}$$

where [I] represents NaR or VR, *K*_i_ is their inhibition constant, “n” is the number of inhibitor molecules that bind the enzyme, “a” is the factor multiplying *K*_i_ that distinguishes the inhibition type, *i.e.* competitive (if a = ∞), purely noncompetitive (if a = 1), or mixed (if a<1 or a>1).

All analyses of best fitting were done by Excel from the indicated number of replicates and, in most cases, we also confirmed the kinetic constants obtained by evaluating X- and Y axis intercepts on corresponding 1/V vs 1/[S] plots.

#### DRG treatment for metabolite extraction, and analysis

Wild-type and *Sarm1*^−/−^ DRG neurons were treated at DIV7 with 3-acetylpyridine (AcPyr) or vehicle (H_2_O), or with vacor or vehicle (H_2_O with 4% 1 N HCl). Proper drug concentrations and times of collection were determined first by dedicated experiments (see Figure 5A). At the indicated time-point, wild-type and *Sarm1*^−/−^ DRG whole explant cultures (typically 30-60 ganglia per condition) were collected, washed in ice-cold PBS and rapidly frozen in dry ice, and stored at −80°C until processed. Next extraction was carried out as reported by grinding in liquid N_2_, resuspending in HClO_4_ containing cAMP as internal standard for recovery calculations, sonicating, and neutralizing with K_2_CO_3_ (Mori et al., 2014). Next HPLC analyses to detect and measure the base exchange formed products, *i.e.* AcPyrAD or the vacorylated compound VAD, were carried out as described above in the paragraph “NADase assays”. In all samples, recovery yields from spiked cAMP were at least 96%. Furthermore, pellets after extraction of soluble nucleotides were treated as reported (Mori et al., 2014) to get reference protein contents to normalize the nucleotide levels. Protein concentrations were determined using the Bradford Reagent (Merck).

#### Neurite degeneration assays in DRG neurons and quantification of degeneration index

Phase contrast images were acquired on a DMi8 upright fluorescence microscope (Leica microsystems) coupled to a monochrome digital camera (Hamamatsu C4742-95). The objective used was HCXPL 20X/0.40 CORR. The degeneration index was determined using a Fiji plugin (Sasaki et al., 2009). For each value, an average was calculated from three fields per condition.

### Quantification and statistical analysis

#### Statistical analysis

One-way ANOVA with Dunnett’s multiple comparison test was performed using R software (https://www.R-project.org/). A p value <0.05 was considered significant and indicated only in most relevant figure data.
