# Supplementary material for: SARM1 is a multi-functional NAD(P)ase with prominent base exchange activity, all regulated bymultiple physiologically relevant NAD metabolites
Source: iScience. 2022 Jan 25;25(2):103812. doi: 10.1016/j.isci.2022.103812 (PMC8844822; doi:10.1016/j.isci.2022.103812)
Supplement: Document S1. Figures S1–S6 and Table S1 [file mmc1.pdf]

iScience, Volume *25*

Supplemental information

SARM1 is a multi-functional NAD(P)ase with prominent base exchange activity, all regulated by multiple physiologically relevant NAD metabolites

Carlo Angeletti, Adolfo Amici, Jonathan Gilley, Andrea Loreto, Antonio G. Trapanotto, Christina Antoniou, Elisa Merlini, Michael P. Coleman, and Giuseppe Orsomando

Fig S1

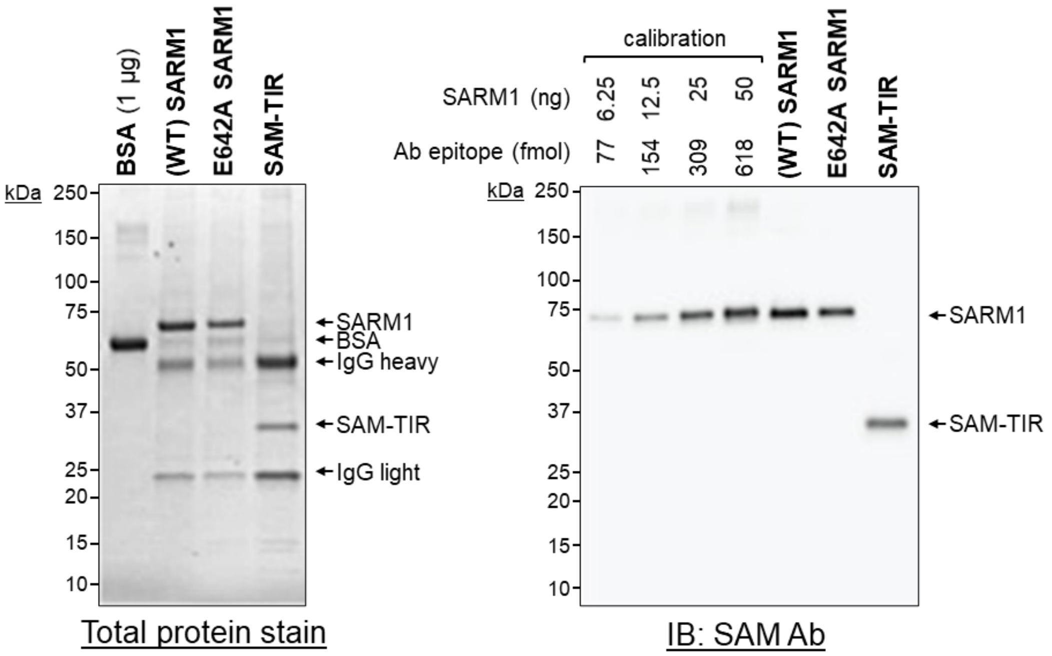

Figure S1. Related to Figure 2 and Figure 3
Purity and quantification of recombinant full length human SARM1 and SAM-TIR fragment.
Representative GelCode™ Blue-stained protein gel (left) and immunoblot probed with a SAM domain antibody (right) of full length WT or E642A human SARM1 and SAM-TIR fragment (aa 409-724) immunoprecipitated from transfected HEK 293T cells. The proteins possess a C-terminal Flag tag and were immunoprecipitated using a Flag antibody as described in Methods. Approximately equimolar amounts of the proteins were run in each case, with approximately 20 times more protein loaded in the left panel for staining. Both full length and SAM-TIR proteins (on beads) were run alongside a dilution series of pure SARM1 standard in solution (not Flag-tagged) to generate a calibration curve to be used for the quantification after probing immunoblots with a polyclonal antibody raised against the SAM domains of SARM1. The predicted MWs of the Flag-tagged proteins (80913.8 for WT full-length and 37237.2 for SAM-TIR) were used to calculate ng equivalents of SAM-TIR versus the concentration calculated for full length SARM1.

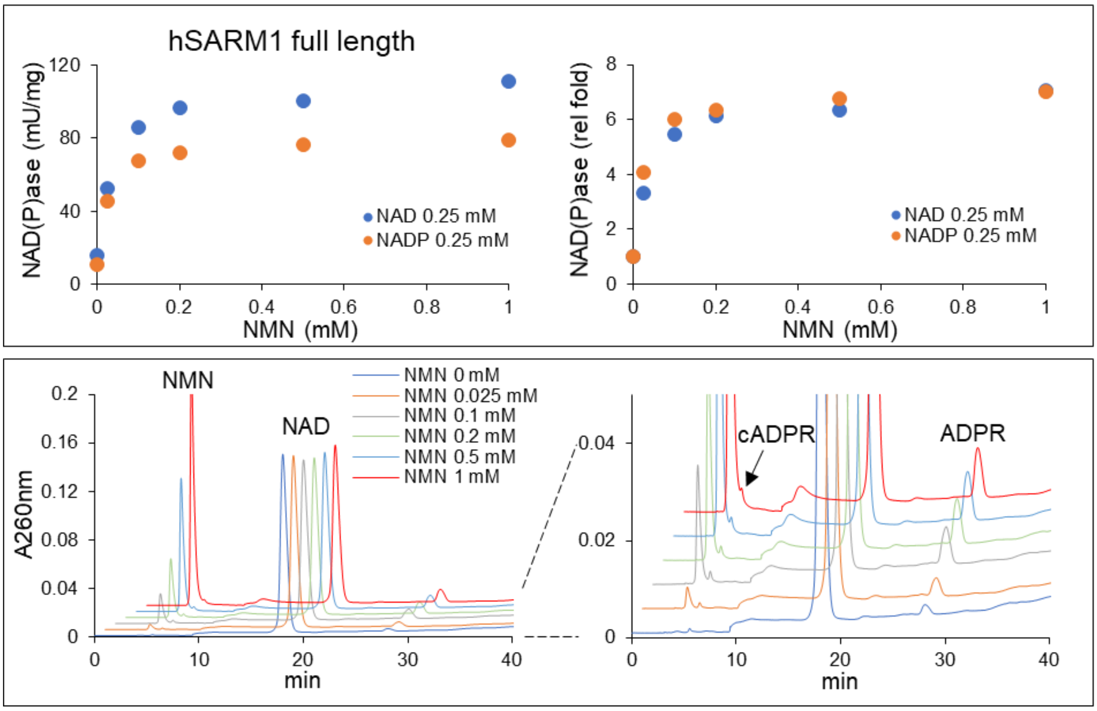

Figure S2. Related to Figure 2 and Figure 3
Human SARM1 NADase and NADPase after induction by NMN
Upper panel, NADase and NADPase activities in comparison of human SARM1 full length (14.6 µg/ml per mix) assayed for 30 min at 25 °C in the presence of variable NMN as indicated (n = 1). Left graph shows absolute rates while relative ones are on the right. Basal rates with NAD and NADP alone at 0.25 mM (activity relative fold = 1) corresponded to 15.8 mU/mg and 11.3 mU/mg, respectively. Bottom panel, C18-HPLC UV profiles of the NAD mixtures above. Only the profiles after 30 min incubation with increasing NMN levels are shown. NMN was not consumed during incubations.

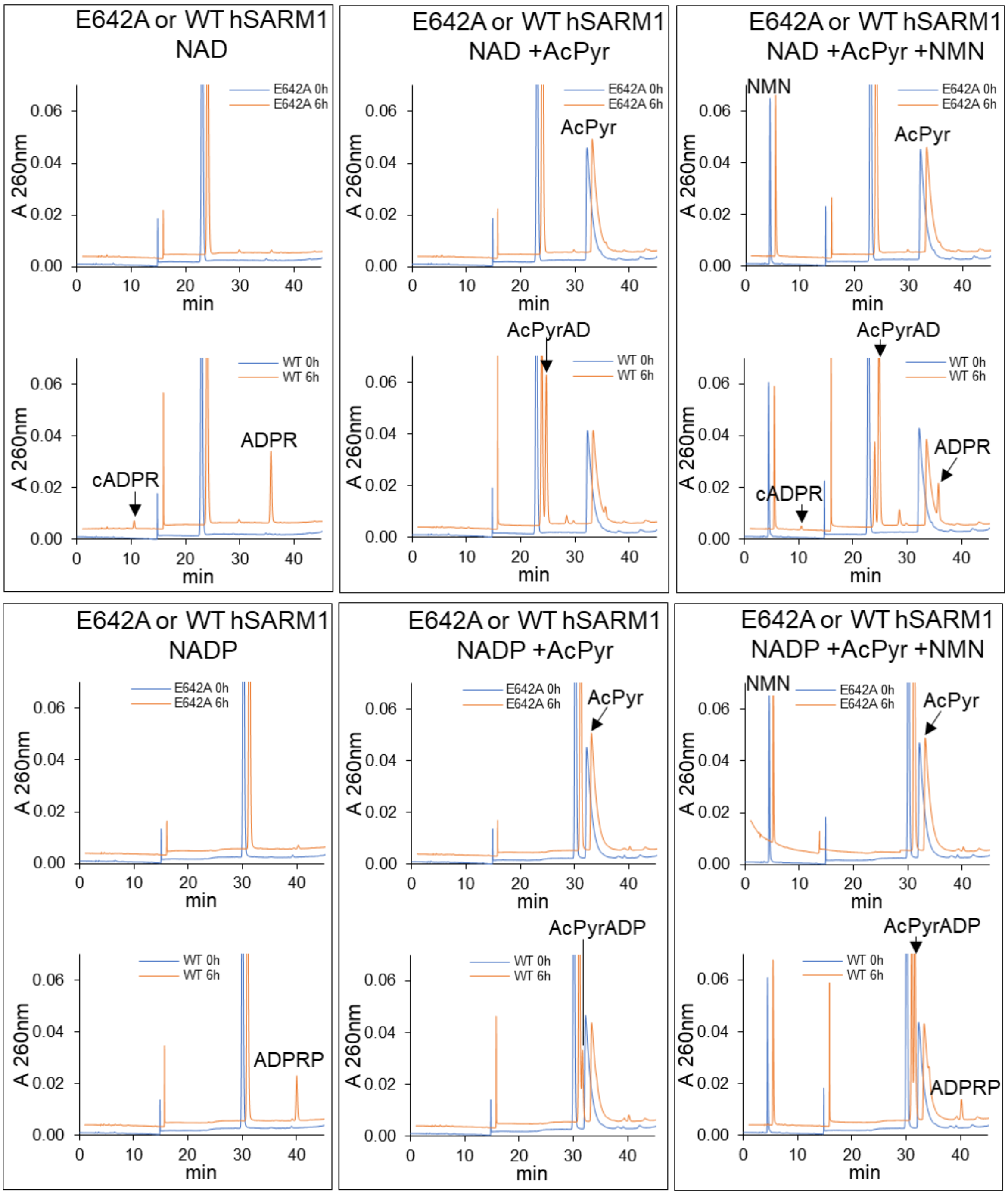

Figure S3. Related to Figure 2 and Figure 4

The human SARM1 mutant E642A lacks any NADase, NADPase, and base exchange activity

NADase or NADPase activities of full length hSARM1 WT (5.4 µg/ml per mix) and those of the mutant E642A (5.6 µg/ml per mix) were assayed for 6 hours at 25 °C (n = 1) in the presence of NAD or NADP 0.25 mM, AcPyr 2 mM, and NMN 0.2 mM as indicated (see titles in individual boxes). The main reactants and products in each case are evidenced. Clearly, the three selected conditions (substrate alone; substrate plus free base; substrate plus free base plus trigger) were to evaluate the multiple activities of SARM1, *i.e.* hydrolysis, cyclization and base exchange, even under NMN triggering effects. Thus, the observed lack of activity of hSARM1 E642 in all such conditions shows the mutated residue is alone crucial to all three catabolic reactions catalyzed by the enzyme (see Fig. 1B). Herein measured basal rates with NAD and NADP alone were 26.4 mU/mg and 15.9 mU/mg, respectively.

Fig S4

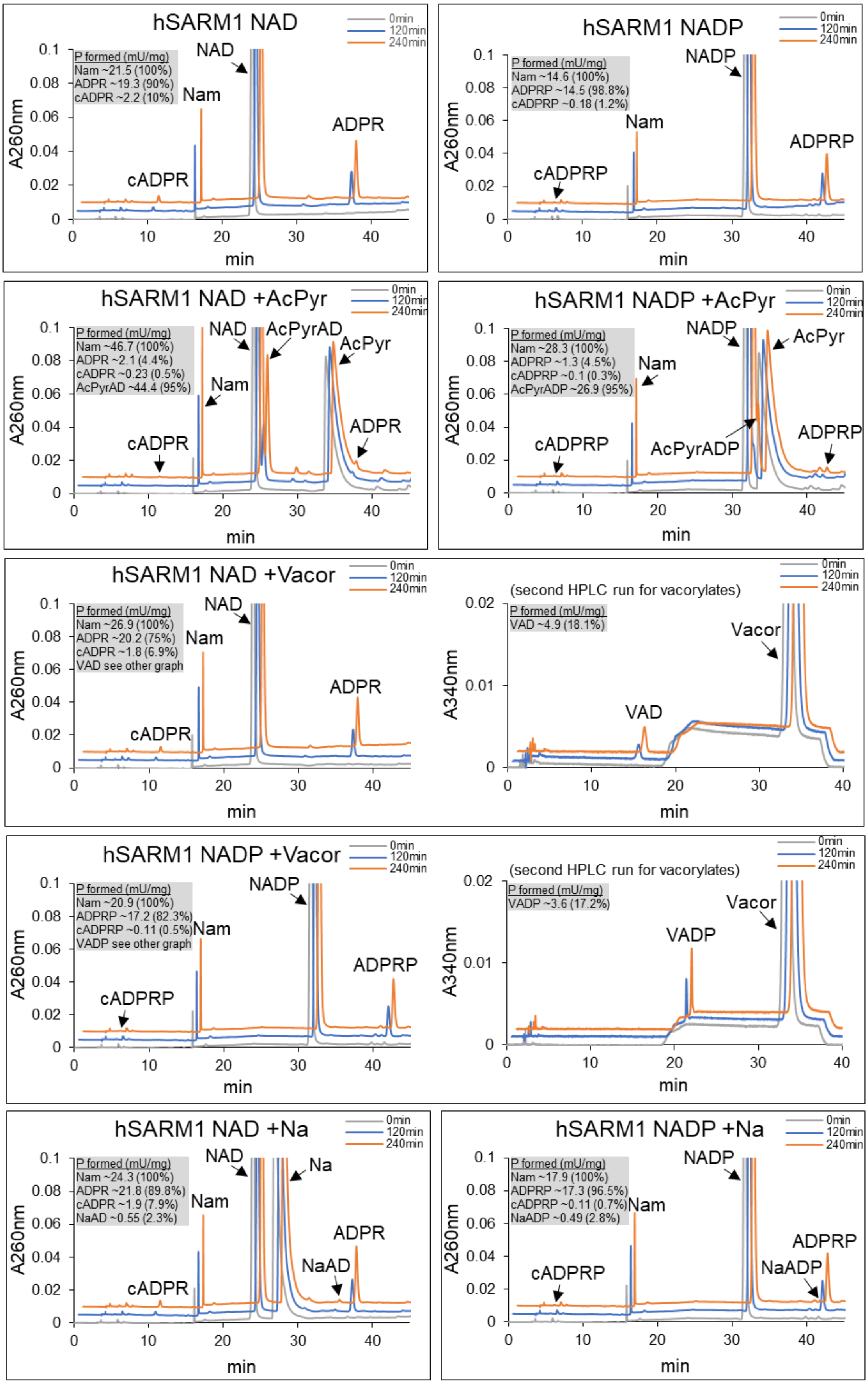

Figure S4. Related to Figure 4

C18-HPLC analysis of typical base exchange reactions catalysed by human SARM1 full length in the presence of single free bases in millimolar excess

Data set of one replicate presented in Fig. 4B left panel (assay conditions are detailed in the legend). C18-HPLC analyses were duplicated for vacor compounds (see Methods). Rates in grey boxes are calculated from the distinct products as indicated, all linearly accumulating by time.

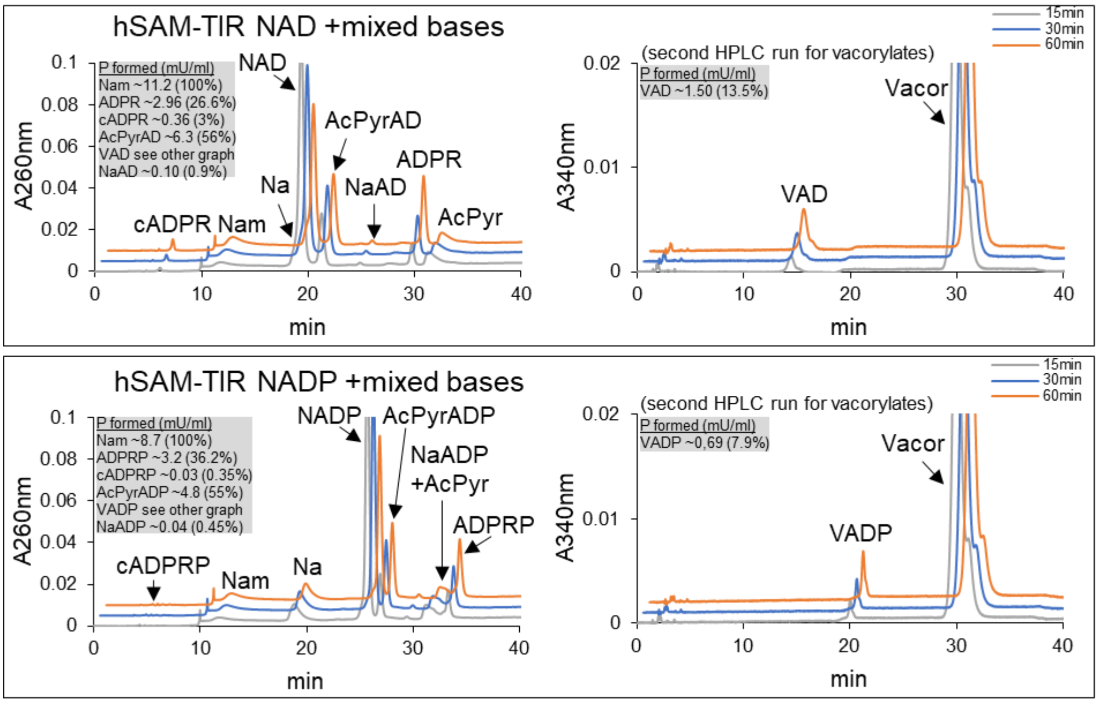

Figure S5. Related to Figure 4

C18-HPLC time course analysis of human SARM1 SAM-TIR base exchange reactions with mixed micromolar concentrations of free bases in the assay mixture

Base exchange reactions at pH 7.5 carried out with ~5 µg/ml SARM1 SAM-TIR and 250 µM NAD or 250 µM NADP plus free bases mixed together, 3-acetyl pyridine (AcPyr), vacor and nicotinic acid (Na), all three at 250 µM final (equivalent to the substrate). Multiple time stops from both mixtures were collected and analyzed by C18-HPLC showing formation of base exchange products as follows: AcPyrAD from AcPyr and NaAD from Na in the presence of NAD (upper left panel); AcPyrADP from AcPyr and NaADP from Na in the presence of NADP (bottom left panel). The vacor-derived dinucleotide analogs VAD and VADP were measured by duplicated C18-HPLC analysis onto a modified method as described in Methods (right panels). Rates of Nam release indicated in grey boxes corresponded in all cases to the whole substrate consumed, and all other products corresponded to relative percentages of this total as displayed. The assay shows that these three bases lead to formation of corresponding base exchange products even all are present. Furthermore, total base exchange by SARM1 SAM-TIR under these conditions was 70.4% with NAD and of 63.4% with NADP, both in keeping with values obtained when individual bases were used in larger molar excess (see Table S1 and Fig. S4). This reinforces regarding dominance and likely physiological occurrence of SARM1-catalysed base exchange reactions.

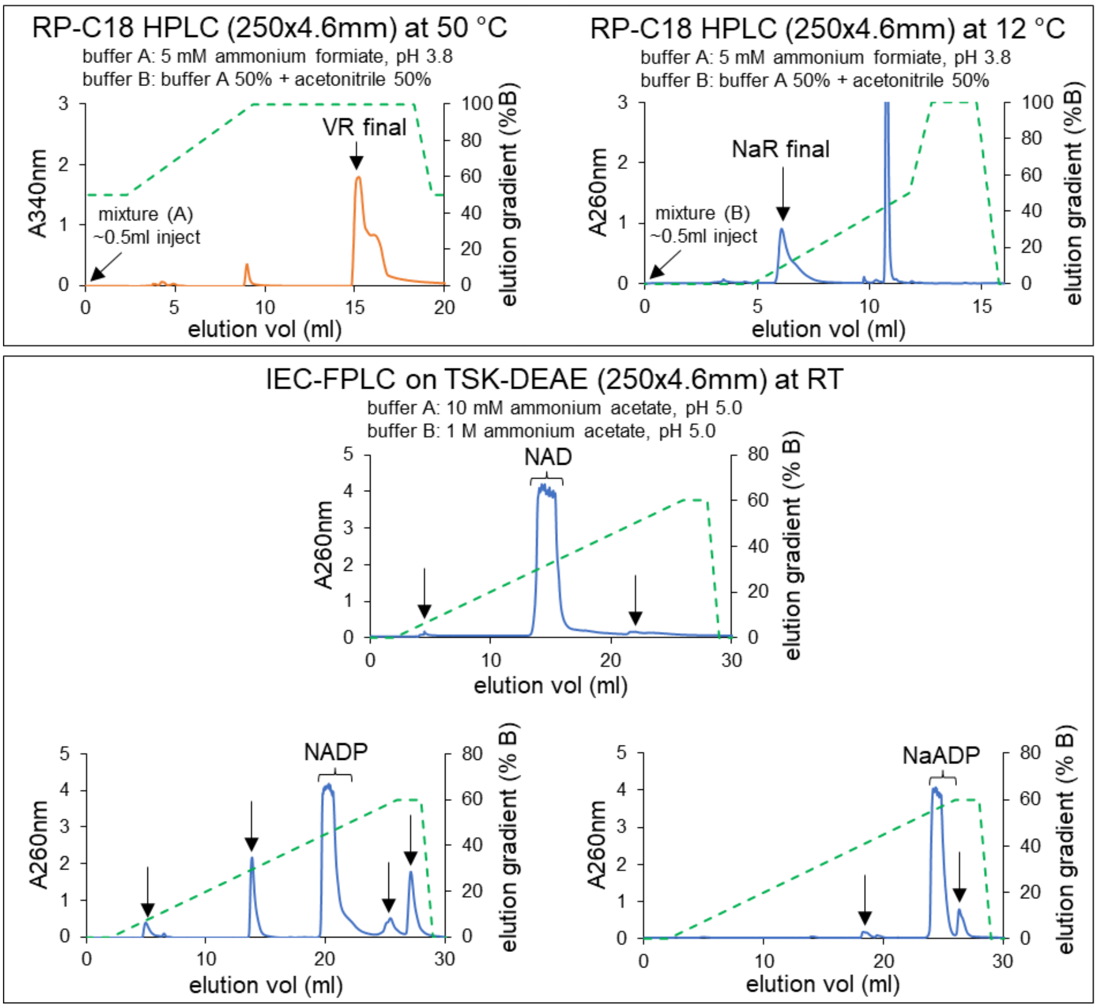

Figure S6. Related to STAR Methods

Preparative chromatographies used to purify VR or NaR, both arising from enzymatical synthesis, or to clean up NAD, NADP, and NaADP

Upper panel, mixtures (A) for VR and (B) for NaR obtained and treated as described in Methods were injected onto a reverse phase C18-HPLC gradient as shown. The peaks indicated by vertical arrows coeluted with VR and NaR pure standards; they were collected and lyophilized.

Bottom panel, commercially available NAD or NADP or NaADP stocks were all purified before usage in subsequent enzymatic assays by IEC-FPLC as shown. The peaks indicated below the parentheses were collected and lyophilized. Black arrows indicate the most frequent contaminants removed.

Table S1. Related to Figure 4

Rates of typical base exchange reactions catalysed by human SARM1 full length, human CD38, and *Aplysia californica* ADP ribosyl cyclase at neutral pH

Data values presented in Figure 4 as histograms are detailed further below (Mean ± SEM, n = 2). The different hSARM1 products (see Fig. 1B) linearly accumulating under conditions that are summarized on top of the table were measured for calculation of hydrolysis (ADPR or ADPRP), cyclization (cADPR or cADPRP) or the various base exchanges (AcPyrAD, VAD, NaAD from NAD or AcPyrADP, VADP, NaADP from NADP, as also shown in Figg. S4 and S5).

	Assay reagents for base exchanges							
NAD 0.25 mM	+	+	+	+	-	-	-	-
NADP 0.25 mM	-	-	-	-	+	+	+	+
AcPyr 2 mM	-	+	-	-	-	+	-	-
Vacor 0.5 mM	-	-	+	-	-	-	+	-
Na 2 mM	-	-	-	+	-	-	-	+
	hSARM1 full length							
rate (mU/mg)	21.8 ±0.30	42.6 ±3.34	29.5 ±2.55	23.3 ±0.83	14.7 ±0.13	26.6 ±1.71	22.9 ±1.68	17.3 ±0.54
rate (relative fold)	1.0 ±0.014	1.95 ±0.153	1.35 ±0.117	1.07 ±0.038	1.0 ±0.009	1.81 ±0.116	1.56 ±0.115	1.17 ±0.037
hydrolysis	90.65%	4.18%	64.25%	90.05%	98.67%	5.96%	76.98%	96.96%
cyclization	9.35%	0.58%	5.58%	7.41%	1.33%	0.14%	0.62%	1.00%
base exchange	-	95.24%	30.17%	2.54%	-	93.90%	22.41%	2.04%
	hSARM1 full length +NMN 0.2 mM							
rate (mU/mg)	187.9 ±26.9	218.5 ±37.4	180.1 ±8.72	201.5 ±27.9	117.3 ±4.72	131.1 ±17.0	134.7 ±5.67	131.9 ±10.5
rate (relative fold)	8.61±1.23	10.02 ±1.71	8.26 ±0.400	9.23 ±1.28	7.98 ±0.321	8.92 ±1.16	9.16 ±0.386	8.98 ±0.711
hydrolysis	90.49%	6.47%	72.08%	90.07%	99.95%	6.80%	76.34%	98.47%
cyclization	9.51%	0.80%	7.06%	8.35%	~0.05%	nd	~0.05%	~0.05%
base exchange	-	92.73%	20.86%	1.58%	-	93.20%	23.61%	1.48%
	hSARM1 full length +VMN 0.05 mM							
rate (mU/mg)	210.1 ±14.4	228.9 ±18.9	206.2 ±1.12	199.0 ±17.6	138.3 ±1.72	140.7 ±13.3	148.5 ±4.61	147.6 ±4.76
rate (relative fold)	9.63 ±0.660	10.49 ±0.867	9.45 ±0.051	9.12 ±0.805	9.41 ±0.117	9.57 ±0.907	10.11 ±0.314	10.04 ±0.324
hydrolysis	91.21%	5.72%	50.27%	91.88%	99.36%	5.82%	63.94%	98.03%
cyclization	8.79%	0.68%	4.75%	6.44%	0.64%	nd%	0.50%	0.69%
base exchange	-	93.61%	44.98%	1.68%	-	94.18%	35.56%	1.28%
	hSARM1 SAM-TIR							
rate (mU/mg)	342.4 ±56.3	427.3 ±76.5	284.8 ±51.1	315.2 ±69.6	274.7 ±49.7	261.7 ±60.9	266.0 ±54.1	242.0 ±37.3
rate (relative fold)	1.0 ±0.164	1.25 ±0.223	0.83 ±0.149	0.92 ±0.203	1.0 ±0.181	0.95 ±0.222	0.97 ±0.197	0.88 ±0.136
hydrolysis	90.02%	2.80%	77.47%	87.54%	99.95%	3.86%	80.11%	96.49%
cyclization	9.98%	0.47%	8.12%	8.67%	~0.05%	nd	~0.05%	~0.05%
base exchange	-	96.73%	14.42%	3.79%	-	96.14%	19.84%	3.46%
	hCD38							
rate (mU/mg)	7798 ±1624	8284 ±992	6818 ±1376	7934 ±1182	3521 ±605	3255 ±787	3496 ±593	3189 ±622
rate (relative fold)	1.0 ±0.208	1.06 ±0.127	0.87 ±0.176	1.02 ±0.152	1.0 ±0.172	0.92 ±0.223	0.99 ±0.169	0.91 ±0.177
hydrolysis	99.38%	93.63%	98.65%	99.41%	99.9%	93.15%	94.45%	99.9%
cyclization	0.62%	0.53%	0.61%	0.59%%	~0.1%	~0.1%	~0.1%	~0.1%
base exchange	-	5.84%	0.75%	nd	-	6.75%	5.45%	nd
	Aplysia cyclase							
rate (mU/mg)	14712 ±399	13996 ±624	12748 ±2593	14595 ±3147	10170 ±232	15779 ±5215	8132 ±2201	9872 ±1846
rate (relative fold)	1.0 ±0.027	0.95 ±0.042	0.87 ±0.176	0.99 ±0.214	1.0 ±0.023	1.55 ±0.513	0.80 ±0.216	0.97 ±0.181
hydrolysis	5.0%	4.60%	7.89%	9.71%	11.22%	6.43%	10.89%	13.82%
cyclization	95.0%	77.88%	86.25%	90.29%%	88.78%	50.30%	67.15%	86.18%
base exchange	-	17.51%	5.86%	nd	-	43.27%	21.96%	nd

Yellow values are fixed on each enzyme species for a relative comparison of the results.
